# Gender differences in global antimicrobial resistance

**DOI:** 10.1038/s41522-025-00715-9

**Published:** 2025-05-19

**Authors:** Mahkameh Salehi, Ville Laitinen, Shivang Bhanushali, Johan Bengtsson-Palme, Peter Collignon, John J. Beggs, Katariina Pärnänen, Leo Lahti

**Affiliations:** 1 Department of Computing, University of Turku, Turku, Finland; 2 Division of Systems and Synthetic Biology, Department of Life Sciences, SciLifeLab, Chalmers University of Technology, Gothenburg, Sweden; 3 Centre for Antibiotic Resistance Research (CARe), Gothenburg, Sweden; 4 Department of Infectious Diseases, Institute of Biomedicine, The Sahlgrenska Academy, University of Gothenburg, Gothenburg, Sweden; 5 Microbiology Department, ACT Pathology, Canberra Hospital, Garran, Australia ACT; 6 Medical School, Australian National University, Canberra, Australia ACT; 7 Independent researcher, Melbourne, Australia VIC

**Keywords:** Metagenomics, Microbiome

## Abstract

Antimicrobial resistance is one of the leading causes of mortality globally. However, little is known about the distribution of antibiotic resistance genes (ARGs) in human gut metagenomes, collectively referred to as the resistome, across socio-demographic gradients. In particular, limited evidence exists on gender-based differences. We investigated how the resistomes differ between women and men in a global dataset of 14,641 publicly available human gut metagenomes encompassing countries with widely variable economic statuses. We observed a 9% higher total ARG load in women than in men in high-income countries. However, in low- and middle-income countries, the difference between genders was reversed in univariate models, but not significant after adjusting for covariates. Interestingly, the differences in ARG load between genders emerged in adulthood, suggesting resistomes differentiate between genders after childhood. Collectively, our data-driven analyses shed light on global, gendered antibiotic resistance patterns, which may help guide further research and targeted interventions.

## Introduction

Antimicrobial resistance (AMR) causes 1.3 million deaths annually^1^, and it has been predicted that AMR will contribute to 39 million deaths from 2025 to 2050^2^. AMR is also one element of the healthy microbiome concept^3^, but despite its relevance to health, little is known about how antibiotic resistance is distributed along socio-demographic gradients, including gender, which could help target interventions. The World Health Organization has called for AMR research that includes gender and socio-demographic considerations and has highlighted the possible increased risk of exposure to AMR that women may encounter^4^.

Previous studies have shown that antibiotic resistance is more prevalent in countries with lower socio-demographic parameters, such as poor infrastructure, low Gross Domestic Product (GDP), and higher levels of corruption^5,6^. However, gender differences in antibiotic resistance have not been studied systematically. In a recent study, we reported that in Finland, a Nordic high-income country, women had a higher average antibiotic resistance load than men across a variety of socio-demographic gradients, including population density, income level, and dietary patterns^7^. This difference between genders may be due to both lifestyle and biological variation^4^. Men and women differ in their predisposition to seek medical care, habitual diets, participation in caretaking, and occupational exposure to resistant bacteria^4,8^. Biological differences include, for example, the propensity for urinary tract infections, childbirth, and hormonal and immunological differences.

This study investigates how the resistome varies between genders along global socio-demographic gradients in a compiled dataset of publicly available shotgun metagenomes from the human gut. We use this data to explore the hypothesis that women carry more ARGs in their gut microbiome than men and that the resistome composition differs between genders. This has implications for women’s health on a global scale. Additionally, we investigate at which age these differences occur and whether country-level socio-demographic gradients are associated with gender-specific variations in the resistome. We investigated the associations in different continents to ensure the robustness of the results. Finally, we describe the overall patterns of resistomes in both genders from infancy to old age, along with global geographic and socio-demographic gradients.

## Results

**Fig. 1:**
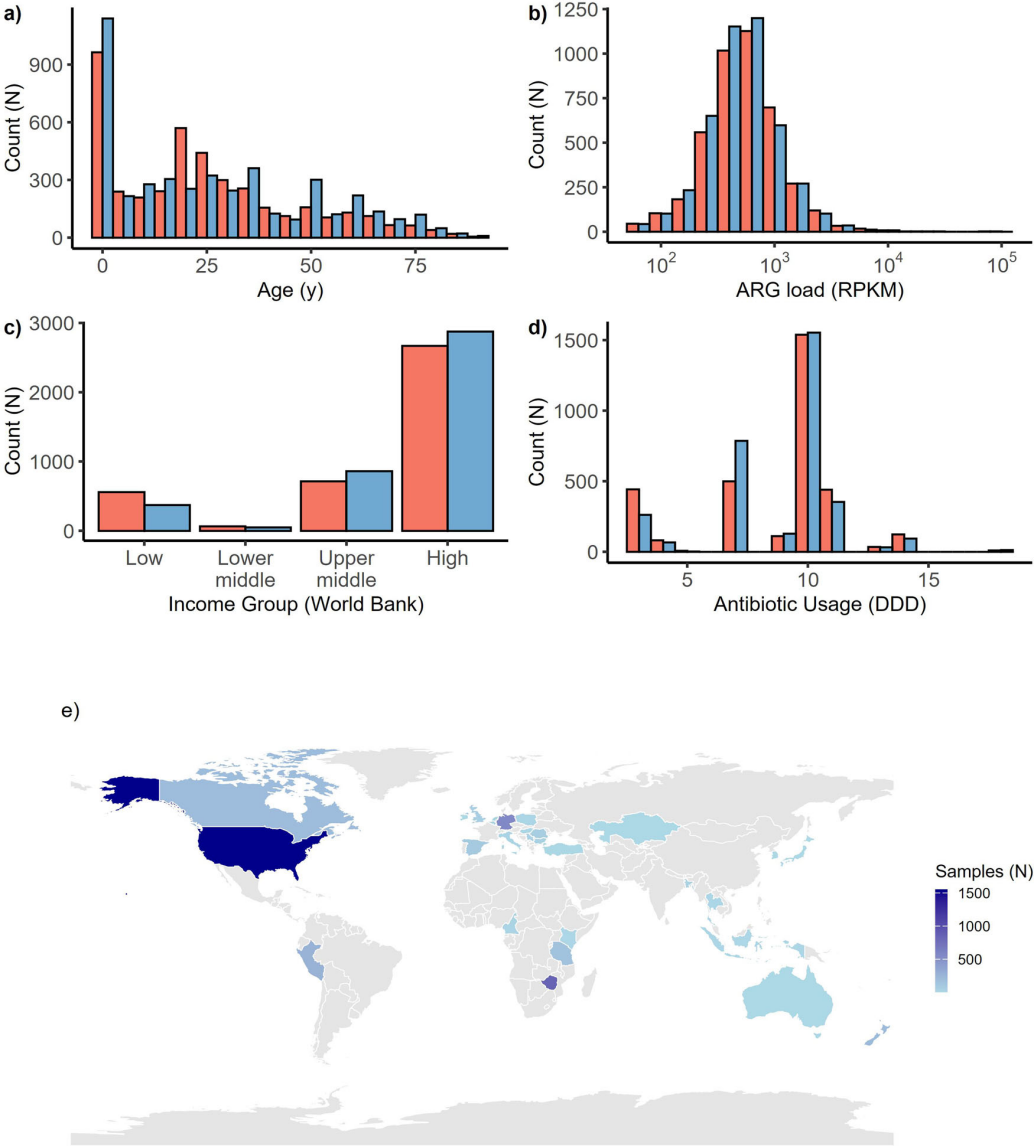
Overview of demographic, economic, and antibiotic resistance data (*N* = 14,641). **a** Age distribution (men: blue, women: red). **b** Antibiotic resistance gene (ARG) load distribution (log RPKM). **c** World Bank income groups (low income, lower-middle income, upper-middle income (low- and middle-income countries, LMICs), and high income (high-income countries, HICs)). **d** Antibiotic use (daily defined doses, DDD per 1000 inhabitants). **e** Geographic distribution of the samples on a world map.

In total, we selected for analysis 14,641 human gut metagenome samples passing quality filtering and having gender information. Figure 1 outlines the distribution of metagenomes by individual age, gender, ARG load, and country-level data, including World Bank income group classification, GDP per capita, and average antibiotic use. The gender distribution was balanced, with a 51%/49% women/men ratio (Fig. 1a). Furthermore, the sample sizes for men and women were similar across all region/age class combinations, both in high-income countries (HIC) and low- and middle-income countries (LMIC) (Supplementary Fig. 1). Thus, we can assume that the observed differences in ARG load and diversity are not confounded by biases associated with uneven demographic distributions. To further assess whether collinearity between predictors could influence our analyses, we conducted a variance inflation (VIF) analysis, which indicated no evidence of collinearity (all VIF values < 5). Of the selected metagenomes, 8,590 (58.7%) had age information, with a median age of 22. The median ARG load was 523 (reads per kilobase per million reads, (RPKM); Fig. 1b). The economic variables and antibiotic data were collected and standardized in a previous publication^5^. High-income countries were overrepresented in the data (Fig. 1c), reflecting the lack of sequencing data from low- and middle-income economies. Median antibiotic use in the countries was 10.3 defined daily doses per 1000 inhabitants (Fig. 1d) The data includes samples from 32 countries, including North America (N = 7882; 53.86%), Europe (N = 2134; 14.58%), Africa (N = 2128; 14.54%), Asia (N = 1053; 7.20%), South America (N = 739; 5.05%), and Oceania (N = 699; 4.78%) (Fig. 1e).

### Overall resistome composition is similar between genders

Resistome composition is a key factor determining which antibiotics it provides resistance to. The overall resistome composition is tightly linked to the taxonomic composition of the microbiome^9^. Beta-diversity provides a measure on how resistomes differ between samples, with a focus on compositional dissimilarities of the ARG profiles. This can provide insights into the broad variation of resistome composition at the population level.

**Fig. 2:**
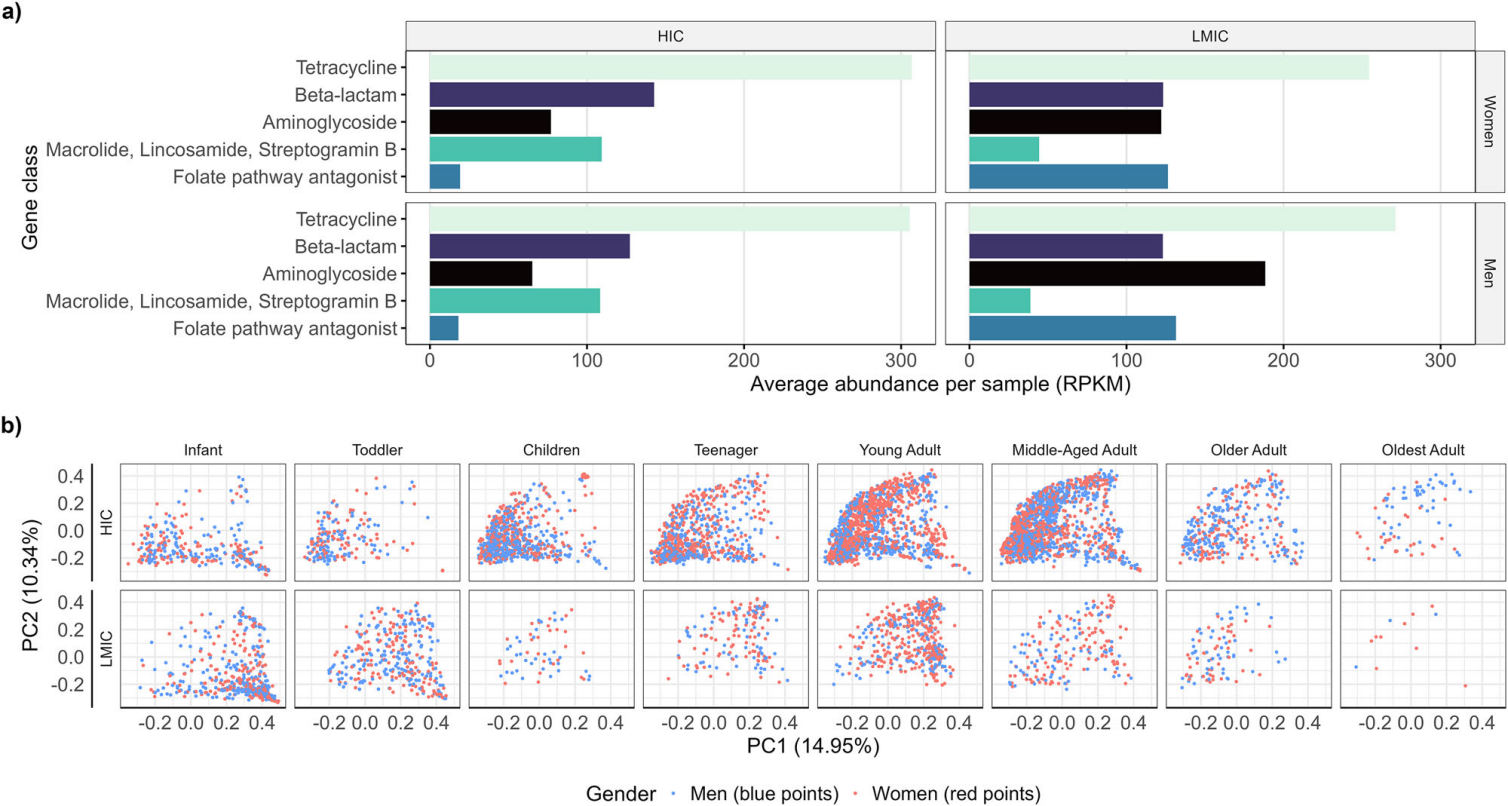
Population variation in resistome composition by age and income groups, and gender. **a** The top panel shows the five most abundant antibiotic resistance gene (ARG) classes across gender and income groups (HIC: *n* = 5544; LMIC: *n* = 2611). **b** Women and men have a similar resistome composition across the different age and income groups (men: blue, women: red). Gender explains only a relatively small, albeit significant, fraction of the overall variation in resistome composition (0.28%; p < 0.001). Principal Coordinates Analysis ordination (PCoA) illustrates the dissimilarity between samples in terms of their overall resistome composition, summarized along the two principal axes (PC1-2; N = 8590 samples with age and gender information, see Supplementary Table 11 for sample sizes; Bray-Curtis dissimilarity index) that explain 14.95% and 10.34% of the overall population-level variation, respectively. The ordination includes all samples, with their distribution along the axes displayed separately for each age-income subgroup. HIC high-income countries, LMIC low- and middle-income countries. For definitions of the age categories, see “Methods”.

ARG classes had distinct prevalence patterns across geographic and demographic groups (Fig. 2a). Analysis of the five most abundant ARG classes revealed that tetracycline resistance genes were the most abundant across all populations, with notably higher levels in high-income countries (HICs) compared to low- and middle-income countries (LMICs). Notable differences between HIC and LMIC populations were observed in aminoglycoside resistance genes and folate pathway antagonist genes: LMIC populations had much higher levels compared to HIC populations.

The variation in resistome composition was significantly associated with gender, age, region, GDP per capita, and antibiotic use (multivariable PERMANOVA, R² = 0.13%–4.9%; p < 0.001 for all variables; Fig. 2; Supplementary Table 1). To further investigate resistome variation, we performed Principal Coordinates Analysis (PCoA) using Bray-Curtis dissimilarity, stratified by gender, region, age category, and income group (Supplementary Fig. 2). While gender explained a small fraction of the resistome variation (R² = 0.28%, p < 0.001), Region and age explained the largest share of the variance: R^2^ = 4.9% and R^2^ = 2.8%, respectively. Gender accounted for only a small (0.28%) albeit a significant fraction of the variance (p < 0.001). Similarly, gender has been repeatedly reported to contribute to much less variation in taxonomic composition than other factors such as diet or ethnicity^10^. The overall resistome composition was similar between genders in all age groups (Fig. 2).

### Women have higher ARG load than men in high-income countries

Our previous research indicated that women have a higher ARG load than men among Finnish adults, although ARG diversity does not differ significantly between sexes^7^. To test this hypothesis further, we investigated gender differences in adults in the global dataset. We compared the ARG load between adult men and adult women in the whole data and found a significant difference between the genders (4% increase, p = 1.6 × 10^–5^ Wilcoxon test). Specifically, the median ARG load (RPKM) for women was 547 and 526 for men. Resistome diversity differed between genders, with women exhibiting a slightly but significantly higher average diversity than men (3% increase, respectively, p = 7.13 × 10^−5^; Wilcoxon test). The median ARG Shannon diversity was 1.99 and 1.90 for adult women and men in the whole data, respectively.

**Fig. 3:**
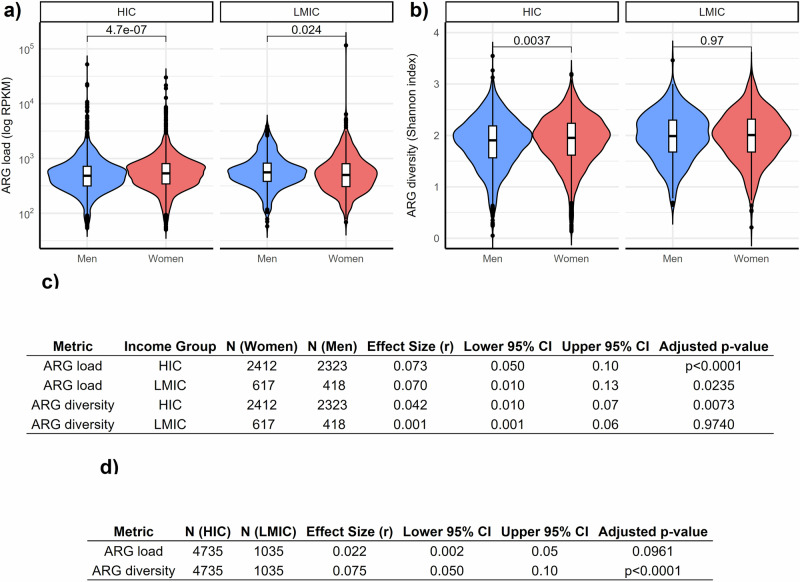
Antibiotic resistance gene (ARG) load and diversity by income and gender. **a** ARG load (RPKM) **b** ARG diversity (Shannon index). The income groups include low- and middle-income countries (LMIC) and high-income countries (HIC) (men: blue, women: red). The boxes indicate medians and interquartile range (IQR) whiskers. The violin plots show the overall population distribution. Statistical significance for group-wise comparisons was determined using the Wilcoxon test. **c** Summary of gender differences in ARG load, and ARG diversity within each income group, including sample sizes, effect sizes (r) with 95% confidence intervals, and adjusted p-values. **d** Summary table reports the Wilcoxon test results for ARG load and diversity by income group, including effect sizes (r) with 95% confidence intervals and adjusted p-values.

Antibiotic resistance may follow different patterns in countries with varying gender norms, occupational distribution, infrastructure, and access to clean water. Therefore, we investigated whether the global average trend differs between low and middle-income countries (LMIC) versus high-income countries (HICs). We discovered that women had a higher ARG load in high-income countries (HICs; 9% increase, Wilcoxon test, effect size = 0.073, 95% CI: 0.050–0.10, p = 4.7 × 10^–7^; Fig. 3a, c). The median ARG load (RPKM) was 560 for women and 515 for men. Interestingly, this pattern was reversed in LMICs, where men had higher ARG load than women (5% increase, p = 0.024). The median ARG load for women was 493 and 517 for men in LMICs.

A similar difference in ARG diversity was observed between men and women in the HICs, with women showing higher diversity on average (Fig. 3b, c; Wilcoxon test, effect size = 0.042, 95% CI: 0.010–0.07, p < 0.0073). We did not observe significant gender differences in diversity in the LMIC group (Fig. 3b, c; effect size = 0.001, 95% CI: 0.001–0.07, p = 0.97).

We also investigated gender differences in countries with low or high antibiotic use (>10 daily defined doses, DDD, per 1000 inhabitants, see Supplementary Fig. 3, and Supplementary Fig. 4) as antibiotic use may differentiate resistance trends. The antibiotic use data corresponds to average use in the country’s whole population and is not stratified by gender. In HICs, regardless of the country’s average antibiotic use, women had higher average ARG loads than men (effect size = 0.133, 95% CI: 0.080–0.18, p = 4.1 × 10^−7^ and effect size = 0.058, 95% CI: 0.030–0.09, p = 0.0019 respectively, for low or high antibiotic use, Supplementary Fig. 3a, e). A reversed trend was observed in LMICs with low average antibiotic use in the country (effect size = 0.065, 95% CI: 0.009–0.13, p = 0.049, Supplementary Fig. 3b, e). In LMICs with high average antibiotic use in the country, there were no significant differences between genders. In both HICs and LMICs, high antibiotic use was associated with higher ARG load (effect size = 0.059, 95% CI: 0.020–0.010, p = 0.0024 and effect size = 0.246, 95% CI: 0.180–0.31, p = 1.1 × 10^−9^ for HICs and LMICs, respectively, Supplementary Fig. 3b, e). ARG diversity was higher in women than men in HICs (p = 0.00042, p = 0.0039, respectively for low or high antibiotic use, Supplementary Fig. 4a, e).

Moreover, the median ARG load and diversity were generally higher in LMICs compared to HICs. Moreover, the median ARG load in LMICs was higher than that in HICs (effect size = 0.022, 95% CI: 0.0016–0.050, p = 0.096) and ARG diversity was significantly elevated in LMICs compared to HICs (effect size = 0.075, 95% CI: 0.05–0.10, p < 0.0001), which might relate to increased spread of ARGs, for example, due to poor sanitation and water quality in LMICs compared to HICs. ARG load was not significantly different in adult women in HICs compared to those in LMICs (p > 0.05, Supplementary Fig. 5). However, ARG diversity was higher in LMIC women compared to HIC women (effect size = 0.061, 95% CI: 0.020–0.10, p = 0.00074, Supplementary Fig. 5).

In addition to the LMIC/HIC categorization, we computed the pairwise differences for ARG load and diversity for adult women and men across regions (Wilcox test, Supplementary Data 2). The regions had significantly different ARG loads and diversities (p < 0.05). ARG load and diversity were highest in Asia for both men and women and regional patterns were similar for both genders. The lowest ARG load and diversity were in Oceania.

### ARG load and diversity are highest in infancy, old age, and women

**Fig. 4:**
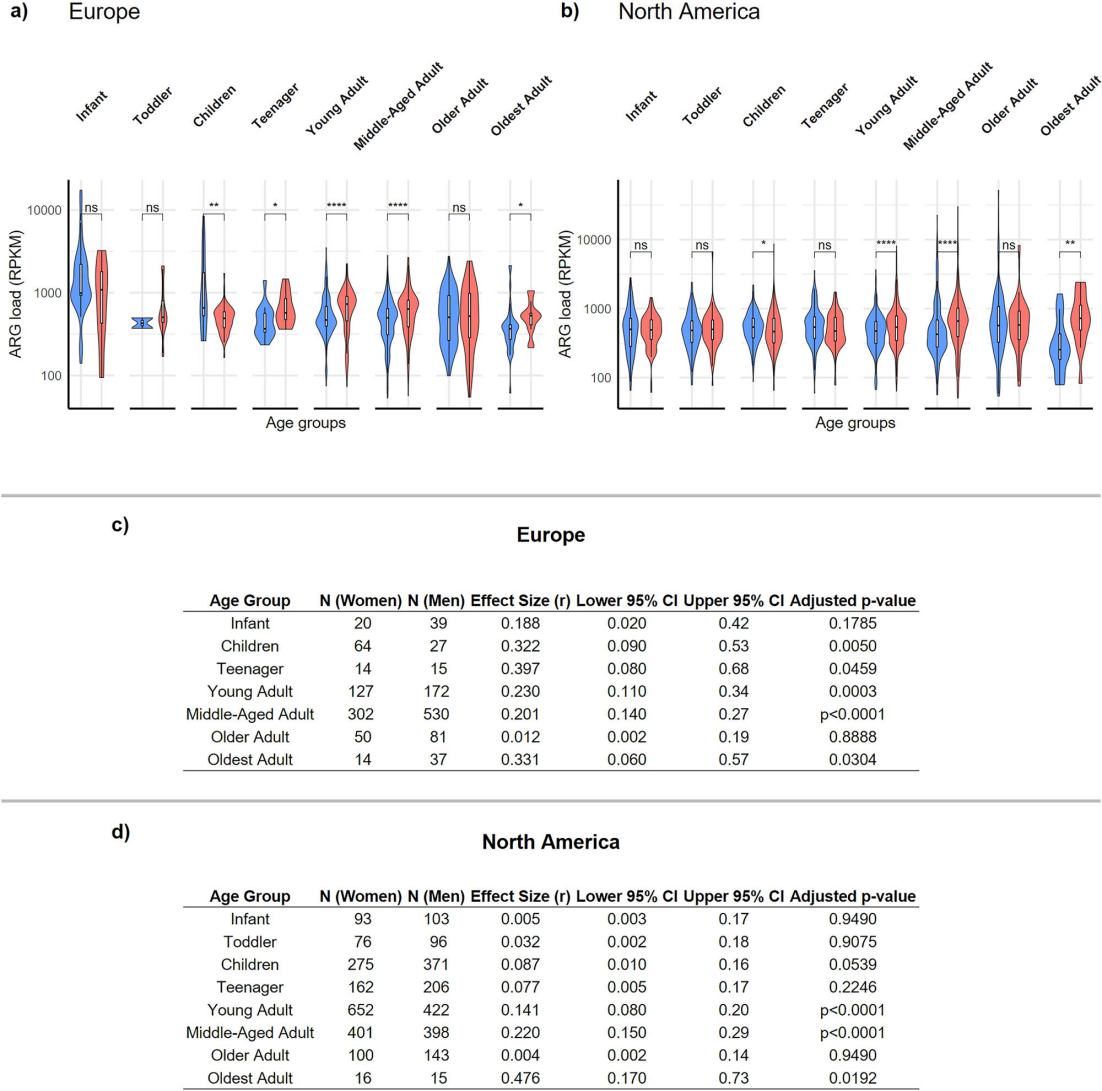
Age, gender, and Antibiotic resistance gene (ARG) load in high-income countries. Gender-specific variation in ARG load from young to old age in high-income countries in **a** Europe and **b** North America. Our data shows that both regions were represented across the entire lifespan. Statistical comparisons of ARG load between genders across age groups (men: blue, women: red), with effect sizes, lower and upper confidence intervals and adjusted p-values, for **c** Europe and **d** North America. The boxes display the median values and interquartile range (IQR) whiskers. For age category definitions, see *Methods*. Statistical significance is denoted as follows: ns: Not significant, p > 0.05. *: p ≤ 0.05, **: p ≤ 0.01, ***: p ≤ 0.001,****: p ≤ 0.0001. Summary statistics for gender comparisons in **c** Europe and **d** North America, including sample sizes, effect sizes (r), and their 95% confidence intervals. Statistical analyses were not performed for age groups with insufficient sample sizes (european Toddler group with only 2 male participants). P-values were adjusted for multiple comparisons using the Benjamini-Hochberg method.

We investigated the trajectory of the resistome from infancy to old age and how gender differences are reflected along age (Fig. 4). Age emerged as a significant factor related to both ARG diversity and ARG load in the human gut microbiome (N = 8590). Women’s ARG load and diversity was significantly higher than men’s, but this difference emerged in adulthood ((HICs), Supplementary Tables 2 and 3; see also Fig. 4, Supplementary Fig. 6). Interestingly, in age group children, males had higher ARG load than females (p < 0.05, Supplementary Tables 2 and 3, Fig. 4, Supplementary Fig. 6). ARG diversity was higher in women in most age categories (Supplementary Fig. 7). In LMICs, the differences between genders in age groups were mostly not significant, possibly due to smaller sample sizes in each age group.

We investigated the trend in ARG load and diversity from infancy to old age. ARG load was highest in infancy and the two oldest age groups in both men and women (Supplementary Data 2). ARG diversity, on the other hand, was highest in infants and toddlers and the oldest adults, and the trend was replicable in both women and men. From the teenage category, the ARG load and diversity progressively increased with age in both women and men.

### Gender, age, and antibiotic use associate with ARG load and diversity

**Fig. 5:**
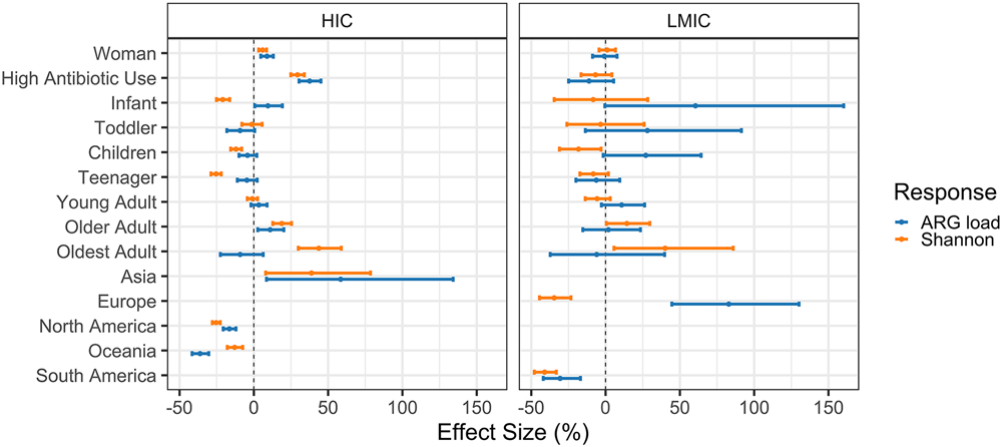
Drivers of antibiotic resistance gene (ARG) load and diversity in high-income countries (HIC) and low- and middle-income countries (LMIC). Probabilistic 95% credible intervals (CI) for the effect size of socio-economic variables on ARG load (blue, modeled using log-normal regression), and Shannon diversity (orange, standard linear regression; see Supplementary Tables 2 and 3). The baseline categories are Europe (in HIC) and Asia (in LMIC), and middle-aged adults for age. The effect sizes were mapped to percentages for easier interpretation, using the transform 100×(exp(*x*) - 1).

We modeled the ARG load and diversity with probabilistic log-normal and standard linear models, respectively, to gain insight into the multivariate associations of socio-demographics (age, gender, region and antibiotic use) with the resistome’s ARG load and diversity (Fig. 5, Supplementary Tables 4–9). We identified these factors as significant predictors of both ARG load and diversity in HICs. Specifically, women had a higher ARG load (8.8% increase, 95% CI = 4.7%–13%, Supplementary Table 4) and higher diversity (5.9% increase, 95% CI = 3.3%–8.5%, Supplementary Table 5). Additionally, higher antibiotic use was positively associated with both ARG load (38% increase, 95% CI = 30%–45%, Supplementary Table 4) and diversity (29% increase, 95% CI = 25%–34%, Supplementary Table 5 in HICs. Samples from HICs in North America and Oceania, had lower ARG load and diversity compared to those from Europe (Supplementary Tables 6 and 7). This pattern was reversed in Asia. Similarly, in LMICs, European and South American samples had lower ARG load and diversity, with the exception of ARG load in European LMICs. Age groups exhibited increase with age for both ARG load and diversity (Supplementary Tables 8 and 9). Samples from Africa, high-income South American countries, and North American and Oceanian low- and middle-income countries had incomplete data for the predictors, and were excluded from the linear models.

We also explored associations between the covariates and the five most prevalent antibiotic resistance classes (Supplementary Fig. 8). In HICs, women had significantly more aminoglycoside and tetracycline resistance compared to men, whilst other classes did not differ statistically significantly. In HICs toddlers, children, and teenagers exhibited higher beta-lactam resistance, and the oldest age groups had elevated amphenicol and aminoglycoside resistance compared to middle-aged adults. High antibiotic use was consistently related to a higher abundance of resistance to all classes of antibiotics. In LMICs, there were no clear trends across antibiotic classes and covariates.

## Discussion

Antimicrobial resistance has been considered as one aspect in the recent efforts to define the concept of healthy microbiome, as resistance in the gut microbiome may elevate the risk of resistant infections^3^. In this study, we explored how the resistome of the human gut microbiome varies by gender along global socio-demographic gradients in 14,641 human gut shotgun metagenome samples from 32 countries. Additionally, we described the trajectory of the resistome in women from infancy to old age. To investigate these differences, we quantified the abundance and diversity of antibiotic resistance genes across diverse populations, stratifying the data by age, geographic region, and economic status. By controlling for potential confounders such as age and region, we identified gender-related patterns in ARG distribution and gained insight into how socio-demographic factors are associated with these trends.

Women had systematically higher average antibiotic resistance loads (9%) and diversity (4%) than men in high-income countries. However, we did not observe significant differences in antibiotic resistance load between genders until early adulthood. In a previous study conducted on adults in Finland, a Nordic high-income country with high socio-economic metrics, we identified that women had, on average, a higher antibiotic resistance load than men^7^. This result was robust to controlling for antibiotic use and other socio-demographic, lifestyle, and health parameters, including diet, income, and taxonomic composition of the gut microbiome. This hints that antibiotic resistance differences may depend on hormonal, immunological, or lifestyle differences that become more pronounced between genders in adulthood.

Differences in the gut resistome composition (beta diversity), which is tightly associated with taxonomic composition, between genders were small but significant (0.28%). Similarly, gender has been reported to have a much smaller effect on taxonomic composition than factors like diet and ethnicity^10^. Amongst all bacterial infections, urinary tract infections (UTIs) are the most common, with women facing up to a 30-fold higher risk compared to men^11^. Women also have a 27% higher likelihood than men of receiving an antibiotic prescription during their lifetime^12^, mainly due to the increased prevalence of UTIs. Additionally, the taxonomic variation between genders^10,13^ may contribute to the observed differences between ARG loads^14^.

In addition to biological differences, lifestyle (including diet, travel, and frequency of person-to-person contact) and occupational differences between genders may predispose women to more risk of acquiring resistant bacteria. Women make up 67% of the frontline healthcare workforce and are more actively involved in teaching and childcare^4^. This increased involvement puts them at a higher risk of infections^4^. Additionally, direct contact with livestock can be a source of resistant bacteria in agricultural and animal husbandry contexts^14^. Women’s level of involvement in these tasks may differ from that of men, depending on the gender norms of the country^4^. For instance, in the EU, more than 90% of childcare workers and teacher aides, as well as nearly 90% of nurses and midwives, were women^15^. In our dataset, information on occupation was not available through the SRA database.

Given that AMR drivers and trends vary across countries with different socio-economics, we also investigated whether HICs and LMICs had differing gender-related trends in ARG load. We observed that the association between ARG load and gender was inverted between HICs and LMICs: women had higher ARG load in HICs and lower ARG load in LMICs than men. However, in multivariate models, no gender difference in LMICs was observed, possibly due to sample size limitations in these countries. This may also be partially explained by the higher rates of antibiotic consumption in women compared to men in HICs^16^, while the opposite trend is observed in low and middle-income settings^17^. Paradoxically, on a global scale, antibiotic use only weakly correlates with antibiotic resistance, and antibiotic resistance levels are exceptionally high in LMICs that use less antibiotics^5^. However, in HIC regions, such as Europe, increased antibiotic use correlates with increased antibiotic resistance^5^. High resistance in LMICs is hypothesized to occur due to increased transmission of antibiotic-resistant bacteria in low-resource settings^18^. In LMICs, presumably, transmission of ARB is a more important driver of AMR than antibiotic use, which is limited by access to antibiotics in these countries^19,20^.

We further investigated the resistome dynamics of resistomes across lifespan, geography, and socio-demographics. We observed that ARG loads were highest in female infants and women over 65, following a U-shape, with a gradual increase from adolescence. The diversity of ARGs followed a similar trend. Similar age trends were also observed in men. Of note, drug resistance in UTIs has been found to increase with advancing age and menopausal status^21^, and infants have been reported to have higher ARG loads than their mothers^22^. Regional differences were pronounced, with individuals from Asia showing the highest Shannon diversity and ARG load in both men and women. Our data did not have enough samples to further stratify the regions, and there are major differences within the regions as well. For example, it is much more prevalent in Bangladesh compared to Japan^23^. However, in women inhabiting high antibiotic use countries (> 10 DDD per 1000 inhabitants), both ARG load and diversity were higher compared to women in low antibiotic use countries (< 10 DDD per 1000 inhabitants). However, due to overlap between region and antibiotic use, some variation between high versus low use may be attributed to other regional factors. Additionally, we observed that the resistome beta diversity was primarily driven by age and geography. Interestingly, age and region explained much more of the variance than gender, which accounted for only 0.28% of the variation between samples.

Our study has several possible limitations. Firstly, metagenomic sequencing can give information on the genetic potential of antimicrobial resistance, but it cannot perfectly predict phenotypic resistance, particularly with short-read sequencing. Secondly, we could not assemble the data due to the data size, varying quality, and sequencing depths, limiting our possibility of assessing the ARGs’ association with mobile genetic elements or the host bacterial species. Thirdly, despite conducting data filtering and manual curation, the metadata retrieved from the public repositories may still be inaccurate. Fourth, the sample collection encompasses a number of different studies from public sources and is subject to various possible biases. For instance, the high-income countries are overrepresented in the data, whereas data from low- and middle-income countries is potentially underpowered, and the age distribution and sample size vary by study and country making stratification by sub-regions difficult. We assessed the observations across different age groups and regions to mitigate these biases. However, such inherent challenges in conducting global studies are difficult to overcome entirely, and there are presently no feasible alternative approaches for such research. In future studies, standardized data collection protocols could help reduce variability. The fifth limitation is that no bacterial species annotation was performed. Therefore, taxonomic differences may contribute to variation in the resistome but are not accounted for. Despite these limitations, the large number of samples provides adequate statistical power to assess these large-scale global patterns using a metric like ARG load, which is more robust to variations in sequencing quality and depth compared to assembly-based methods, and can provide an alternative data source to complement isolation-based AMR surveillance data.

Taken together, our results identify age, gender, region, and antibiotic use as significant factors associated with both ARG diversity and ARG load in the human gut microbiome. Specifically, ARG load and diversity increased with adulthood and were also high in early childhood. Interestingly, ARG loads were weakly but significantly higher in men in LMICs compared to women, potentially reflecting the trends of gendered antibiotic use in HICs and LMICs^16,17^, or the limited sample size in our data from LMICs. Antibiotic use was positively associated with both ARG load and diversity in HICs and with ARG load in LMICs, underscoring its role in shaping resistome characteristics. However, it is clear that this is only one of several factors shaping the human gut resistome. Specifically, these results emphasize the interplay between socio-demographic factors and antimicrobial resistance, highlighting the need for targeted interventions based on age, gender, and regional contexts. Despite the complexity of underlying biological causes, knowing how AMR varies by gender on a global scale can help inform such interventions and further research. Our study demonstrates that gender, life stage, and socio-demographics are interlinked in AMR. Our research may promote awareness of patterns in AMR to guide further research to decipher the causes of gendered risk related to AMR, reduce gender-related AMR mortality, and improve women’s health in the following decades.

## Methods

### Data acquisition from sequence read archive

We utilized BigQuery v2.0.97 to search the nih-sra-datastore.sra.metadata for human gut metagenome accessions. The search parameters included organism = “human gut metagenome”, librarysource = “METAGENOMIC”, assay_type = “WGS”, libraryselection = “RANDOM”, and librarylayout = “PAIRED”. The query retrieved Sequence Read Archive (SRA) metadata for each accession, including country, continent, platform, BioProject, avgspotlength, mbases, and the attributes containing gender and age information. The resulting accessions with metadata on gender or gender were downloaded from SRA using fasterq-dump v3.0.0 and Kingfisher v0.4.1. BioProject summaries were retrieved via Entrez and categorized into groups Infant, Infection, Cancer, Immune Deficiency, Other Diseases, and Others using a custom Python script. The search for accessions was done on June 12th, 2024. Age was converted to years if reported as other units.

### Country-level data for socio-demographic markers

The economic variables GDP per capita, World Bank income class, and antibiotic use data were collected in a previous publication. Antibiotic consumption data were obtained from the IQVIA MIDAS database (Danbury, CT, USA). The GDP per capita was standardized to mean 1 and SD 1 across the countries as described in Collignon et al. 2018^5^.

### Antibiotic resistance gene mapping

We mapped forward reads to the ResFinder database v2.1.1^24^ with KMA v1.4.12a^25^ using Snakemake v7.17^26^ as the workflow manager with the following KMA options: -nc -na -nf -ef -1t1 -ID 80 -ml 60, ensuring each read had a single match with at least 80% sequence identity over a minimum of 60 base pairs. Post-mapping, read counts from KMA mapstat result files were aggregated into a table containing accessions and the number of reads mapped to each gene in ResFinder, using a custom shell script.

### Normalization and ARG metrics

The read counts for each ARG were normalized using the RPKM metric. This involved dividing the number of reads mapped to the ARG by gene length in kilobases and the total number of mapped reads in millions. Gene lengths were sourced from the ResFinder database v2.1.1^24^, and read counts were obtained from the KMA^25^ mapping results.

### Quality filtering

We filtered the data to only include samples sequenced on the Illumina platform with > 1 million reads, an ARG load > 50 RPKM, and > 1 ARG variant observed. In addition to the sequencing depth (read counts), we filtered by minimum ARG load and observed ARGs to ensure that 16S rRNA gene amplicon studies would not be included if they had mislabeled metadata in SRA. After filtering, the read counts were not correlated with ARG load (linear regression, estimate = -0.30, p = 0.81, n = 14,641). Read count was correlated with ARG Shannon diversity (estimate = 0.00938, p < 0.001, n = 14,641) and included as a covariate in the ARG diversity models.

### Statistical analysis

We categorized subject age data in the human gut metagenome samples into eight distinct groups to facilitate stratified analysis: Age categories: Infant [0, 1], Toddler (1, 3], Children (3, 12], Teenagers [12, 20) Young Adult [20, 35), Middle-Age Adult [35, 65), Older Adult [65, 80), and Oldest Adult [80, 100].

This categorization was implemented to align with developmental and lifestyle stages relevant to variations in microbiome composition and ARG load. Sample-specific ARG diversity was quantified using the Shannon diversity index. Exploratory data analysis involved visualizing the ARG load and diversity across all samples. We employed non-parametric Wilcoxon rank-sum tests to identify significant differences between groups in Shannon diversity and ARG load. We utilized the wilcox.test function in R to perform these comparisons. The non-parametric Wilcoxon test was chosen due to the non-normal distributions of some of the key variables. The p-values were corrected for multiple testing using the Benjamini-Hochberg correction. We set the statistical significance threshold for the adjusted values to p = 0.05.

In order to examine the relationship between selected metadata variables and ARG load and diversity, we employed Bayesian log-normal and standard linear regression using the brms package in R. We used age category, gender, low/high antibiotic use, region and HIC/LMIC as predictors. Incorporating age as a predictor ensures that variations in ARG load and diversity are analyzed while accounting for age-related biases across regions. We also tested linear models including log read count as a covariate and a random intercept for study accession number to account for between-study differences (Supplementary Fig. 9). These adjustments did not qualitatively alter the results. Moreover, we used one-hot encoding on the chosen predictors, which results in a separate binary variable for each factor level. Weakly informative N(0,1) priors were assigned to all model parameters, allowing the data to predominantly determine the posterior distributions. In order to quantify the effects implied by the Bayesian models, we used posterior means and quantile-based 95% credible intervals.

Multivariate comparisons conducted using multivariable Permutational Multivariate Analysis of Variance (PERMANOVA). This analysis assessed the overall effect of gender, age category, region, GDP per capita, and antibiotic use on resistome composition based on Bray-Curtis dissimilarity. To ensure that significant PERMANOVA results were not confounded by differences in group dispersions, homogeneity of dispersion test (betadisper) was performed, confirming no significant differences in dispersion between groups (p > 0.05).

Multicollinearity among predictors in the probabilistic regression models was evaluated using Variance Inflation Factor (VIF) analysis. All VIF values remained below the commonly used threshold of 5, indicating that multicollinearity was not a significant concern within the models (Supplementary Table 10).

## Supplementary information

npj_Supplementary

Supplementary_Data1

Supplementary_Data2

## Data Availability

The sequencing data used in the project is openly available from SRA and European Nucleotide Archive (ENA). The metadata table including the variables used for this study is openly available as Supplementary material.

## Code availability

Code is made available in a GitHub repository 10.5281/zenodo.14909582.
