# Supplementary material for: Gender differences in global antimicrobial resistance
Source: NPJ Biofilms Microbiomes. 2025 May 19;11:79. doi: 10.1038/s41522-025-00715-9 (PMC12089330; doi:10.1038/s41522-025-00715-9)
Supplement: Supplementary file 1 — npj_Supplementary [file 41522_2025_715_MOESM1_ESM.pdf]

Supplementary Information

Supplementary information includes:

Supplementary Figures 1-9

Supplementary Tables 1-13

Supplementary Data 1: Accessions and metadata for study
This file contains accession numbers for SRA and ENA metagenomes used in this study, metadata for samples (Age, Gender) and country-level metadata. The file also includes ARG load and ARG diversity calculated for this study

Supplementary Data 2: Pairwise Wilcox tests for regions and age groups (ARG load and diversity).
Sample sizes are given in columns N1 and N2 for Group 1 and 2, respectively. Statistical significance is denoted as follows: **ns**: Not significant, $p > 0.05$. *: $p \leq 0.05$, **: $p \leq 0.01$, ***: $p \leq 0.001$,****: $p \leq 0.0001$.

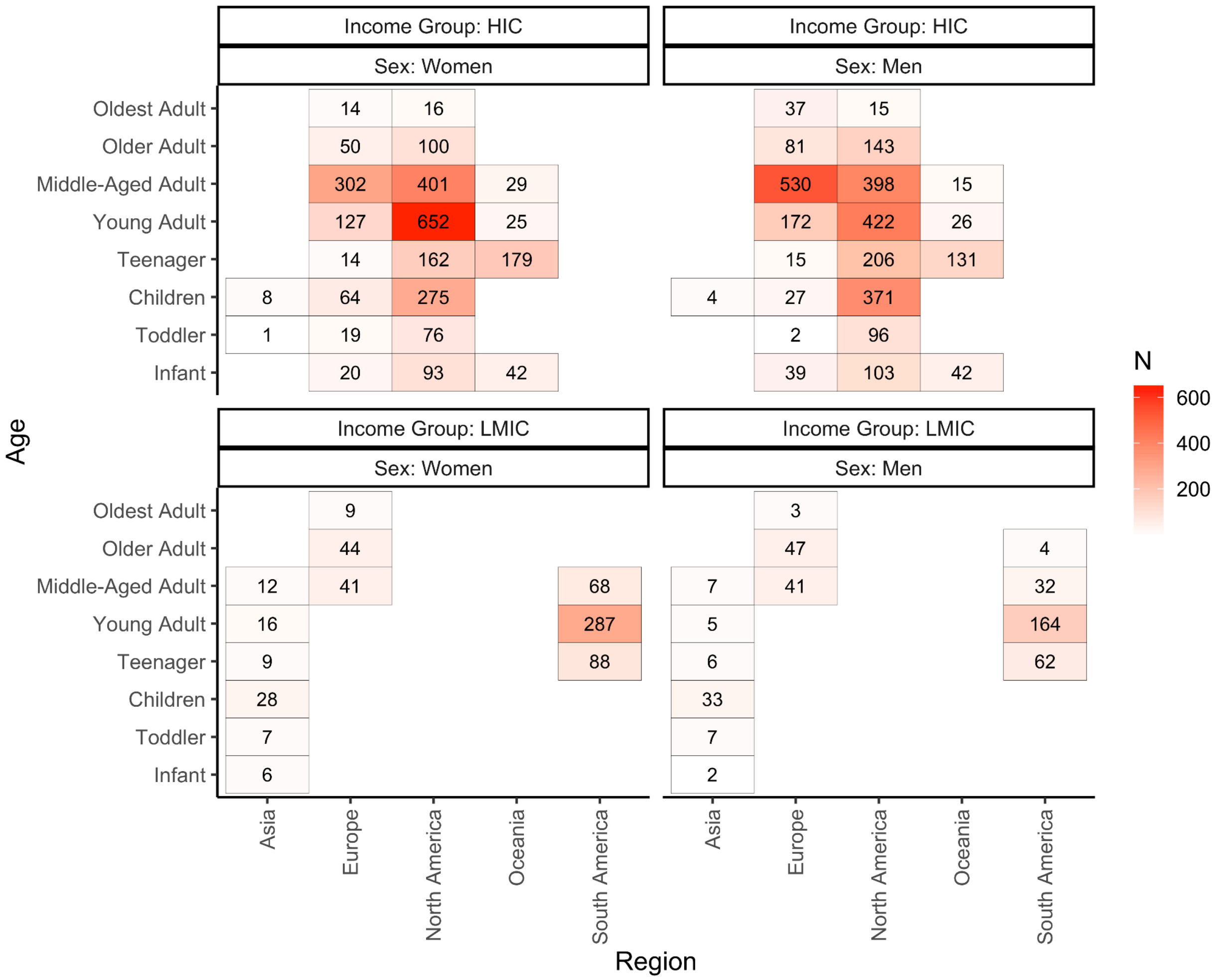

Supplementary Figure 1: Sample sizes across different combinations of age, region, sex, and income level. This heatmap displays the number of participants (N) grouped by age, region, sex, and income level. The top section presents data from high-income countries (HIC), while the bottom section shows data from low- and middle-income countries (LMIC). Each section is divided by gender. The x-axis shows regions (Asia, Europe, North America, Oceania, and South America), and the y-axis shows age groups (Infant, Toddler, Children, Teenager, Young Adult, Middle-Aged Adult, Older Adult, and Oldest Adult). The number of samples (N) is indicated in color (from white (low N) to red (high N)).

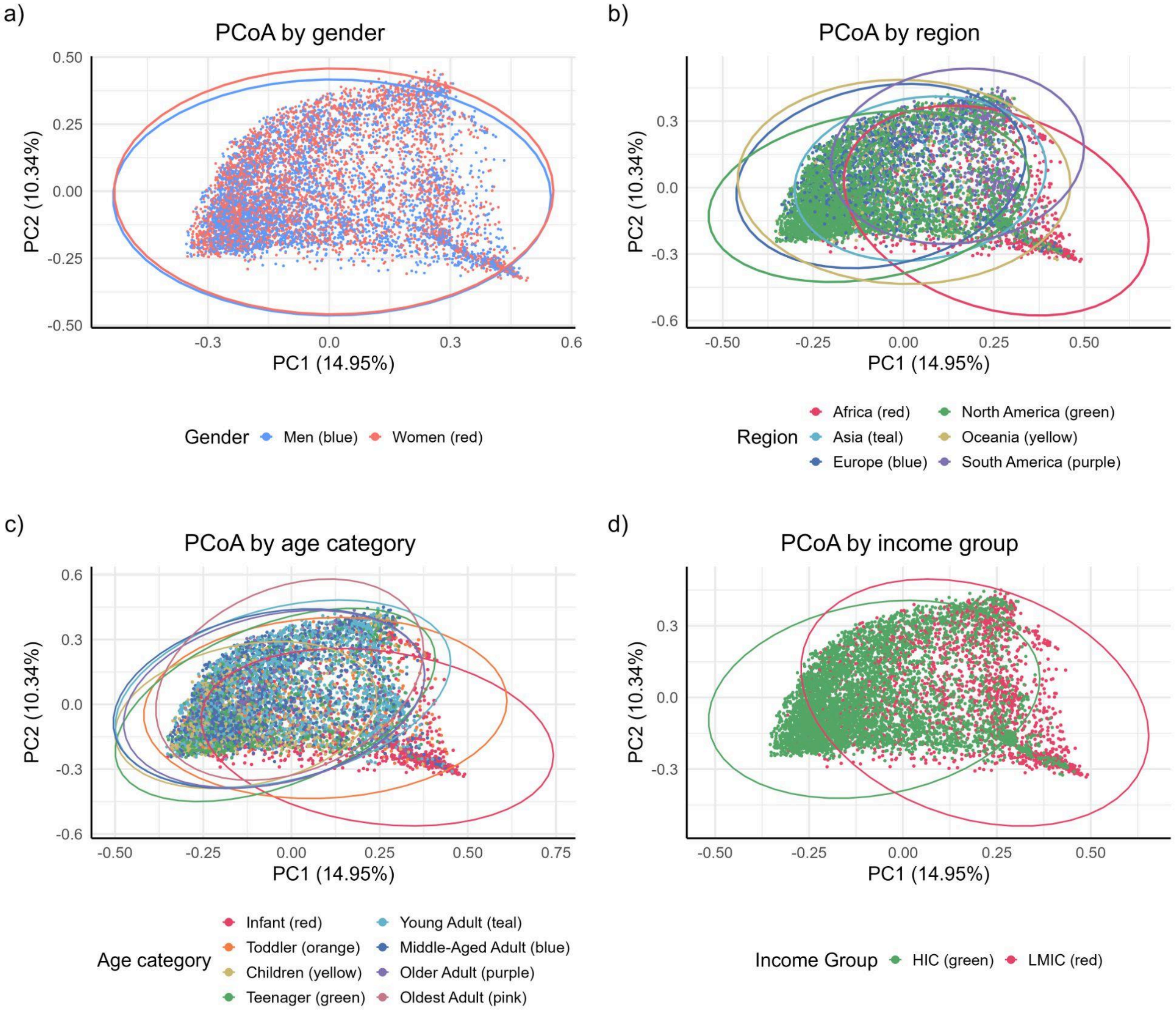

Supplementary Figure 2: Population variation in resistome composition by key demographic and socioeconomic factors. PCoA plots based on Bray-Curtis dissimilarity of samples, with points representing individual samples. Panels illustrate variation in resistome composition when colored by **a)** gender, **b)** geographic region, **c)** age category, and **d)** income group. Ellipses represent 95% confidence intervals for each group.

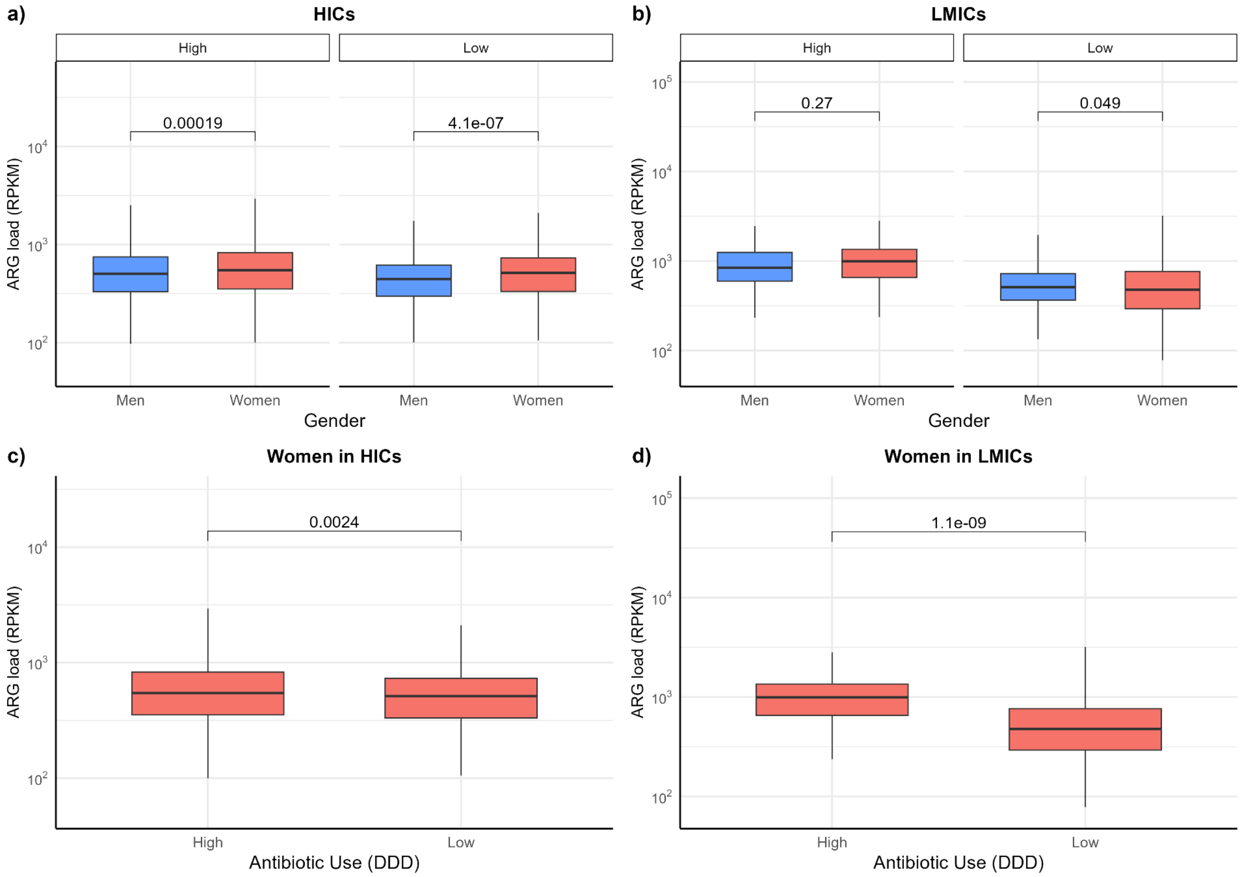

e)

Comparison	Group	N (Group 1)	N (Group 2)	Effect Size (r)	Lower 95% CI	Upper 95% CI	Adjusted p-value
Usage (High vs Low)	HICs	4084	1460	0.100	0.080	0.12	p<0.0001
Usage (High vs Low)	LMICs	102	926	0.242	0.190	0.29	p<0.0001
Usage (High vs Low)	Women in HICs	2098	571	0.059	0.020	0.10	0.0024
Usage (High vs Low)	Women in LMICs	46	569	0.246	0.180	0.30	p<0.0001
Gender (Women vs Men)	HICs - High Usage	2098	1986	0.058	0.030	0.09	0.0002
Gender (Women vs Men)	HICs - Low Usage	571	889	0.133	0.080	0.18	p<0.0001
Gender (Women vs Men)	LMICs - High Usage	46	56	0.110	0.006	0.30	0.2686
Gender (Women vs Men)	LMICs - Low Usage	569	357	0.065	0.009	0.13	0.0488

Supplementary Figure 3. Relationship between Antibiotic resistance gene (ARG) load, gender and country-level antibiotic use in adults. a) Gender differences in ARG load along low (<10 Defined daily doses, DDD per 1000 inhabitants) and high (>10 DDD per 1000 inhabitants) country-level antibiotic use in high-income countries (HIC) **b)** Gender differences in ARG load along low and high country-level antibiotic use in low- and middle-income countries (LMIC) **c)** Difference between ARG load in women in low and high antibiotic use in HIC **d)** Difference between ARG load (log natural) in women in low and high antibiotic use in LMIC. **e)** Statistical summary table reporting sample sizes, effect sizes (r), 95% confidence intervals, and adjusted p-values (Wilcoxon test with Benjamini–Hochberg correction) for the comparisons.

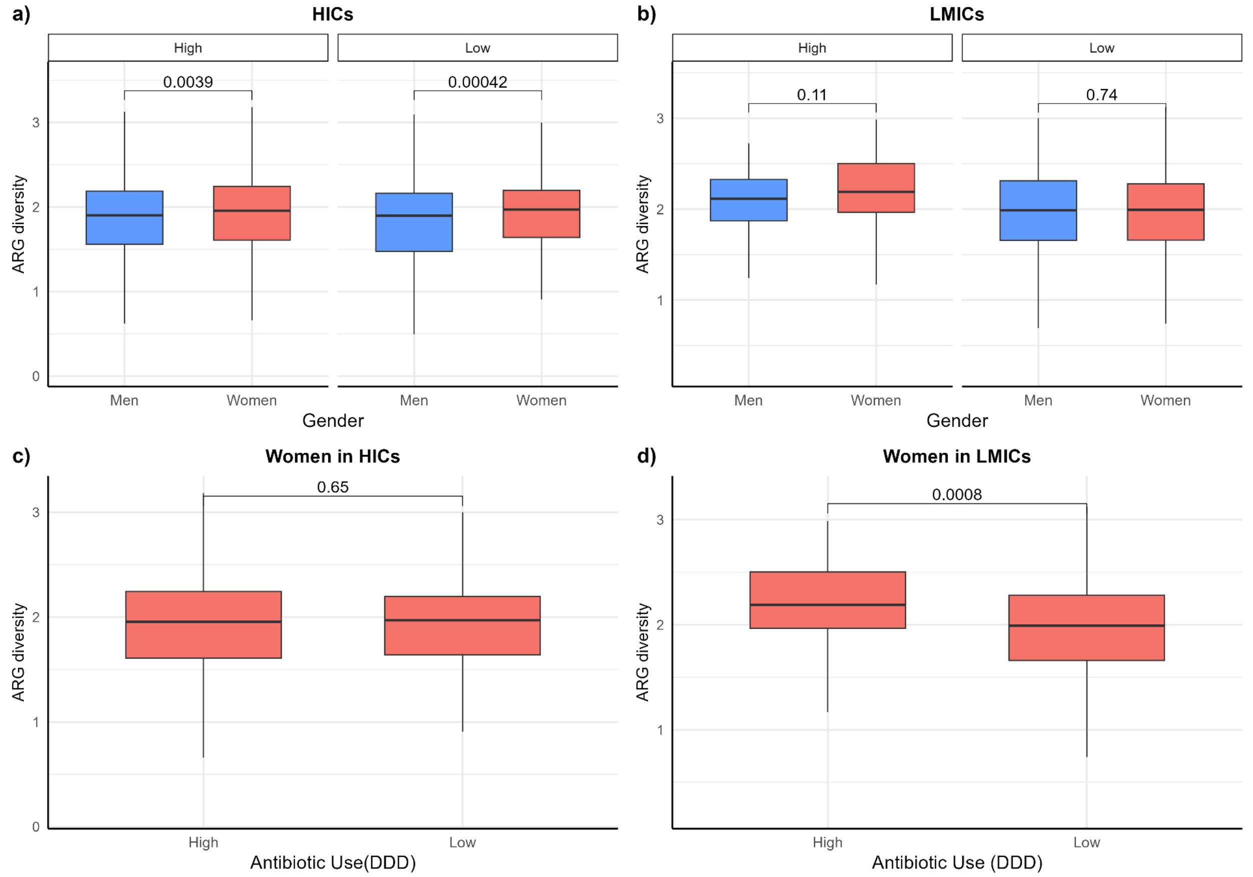

e)

Comparison	Group	N (Group 1)	N (Group 2)	Effect Size (r)	Lower 95% CI	Upper 95% CI	Adjusted p-value
Usage (High vs Low)	HICs	4084	1460	0.019	0.001	0.05	0.1660
Usage (High vs Low)	LMICs	102	926	0.103	0.050	0.16	0.0010
Usage (High vs Low)	Women in HICs	2098	571	0.009	0.001	0.05	0.6517
Usage (High vs Low)	Women in LMICs	46	569	0.135	0.060	0.21	0.0008
Gender (Women vs Men)	HICs - High Usage	2098	1986	0.045	0.010	0.08	0.0039
Gender (Women vs Men)	HICs - Low Usage	571	889	0.092	0.040	0.14	0.0004
Gender (Women vs Men)	LMICs - High Usage	46	56	0.158	0.010	0.34	0.1117
Gender (Women vs Men)	LMICs - Low Usage	569	357	0.011	0.001	0.08	0.7392

Supplementary Figure 4. Antibiotic resistance gene (ARG) diversity across antibiotic use and gender in adults. a) ARG diversity by antibiotic use and gender: Box plots of ARG load across in high-income countries (HICs) for men and women, showing medians and interquartile range (IQR) whiskers. **b)** ARG diversity by antibiotic use and gender in low- and middle-income countries (LMICs). **c)** ARG diversity in women across antibiotic use in HICs and **d)** LMICs. **e)** Statistical summary table reporting sample sizes, effect sizes (r), 95% confidence intervals, and adjusted p-values (Wilcoxon test with Benjamini–Hochberg correction).

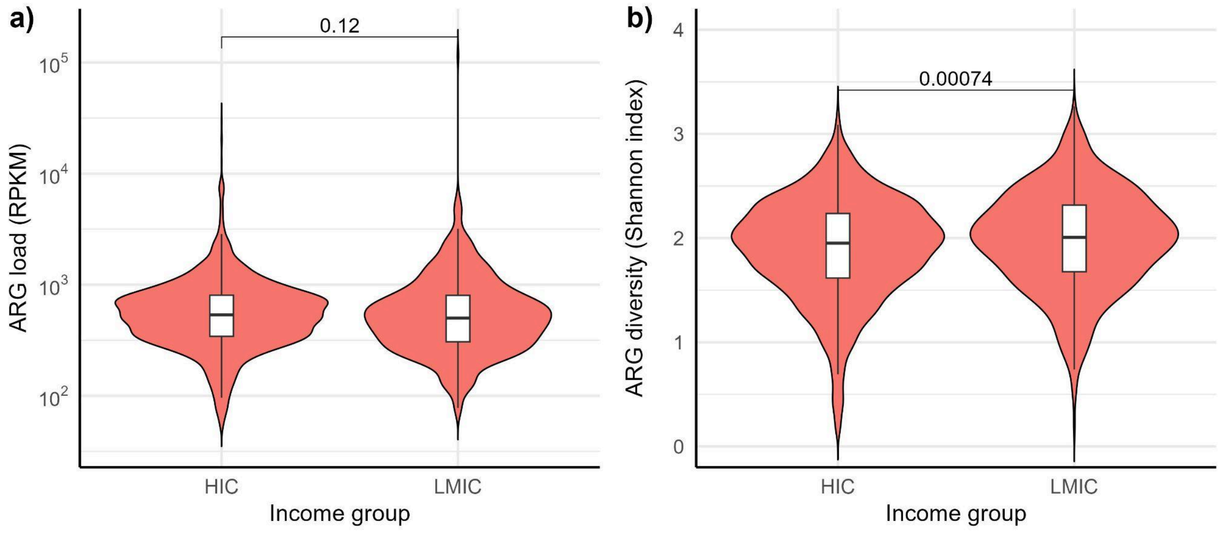

c)

Metric	N (HIC)	N (LMIC)	Effect Size (r)	Lower 95% CI	Upper 95% CI	Adjusted p-value
ARG load	2412	617	0.028	0.002	0.06	0.12207
ARG diversity	2412	617	0.061	0.020	0.10	0.00074

Supplementary Figure 5. Antibiotic resistance gene (ARG) load and diversity across country-level income groups in adult women. The violin plots illustrate the distribution of values, with overlaid boxplots indicating the median and interquartile range (IQR). **a)** Shows ARG load (RPKM) on a log-transformed scale, while **b)** presents ARG diversity using the Shannon index on a linear scale. Statistical comparisons were conducted using Wilcoxon tests with Benjamini–Hochberg adjustment for multiple testing. **c)** presents a summary table reporting, for each metric, the sample sizes for high-income countries (HIC) and low- and middle-income countries (LMIC), the effect size (r) with its 95% confidence interval, and the adjusted p-value.

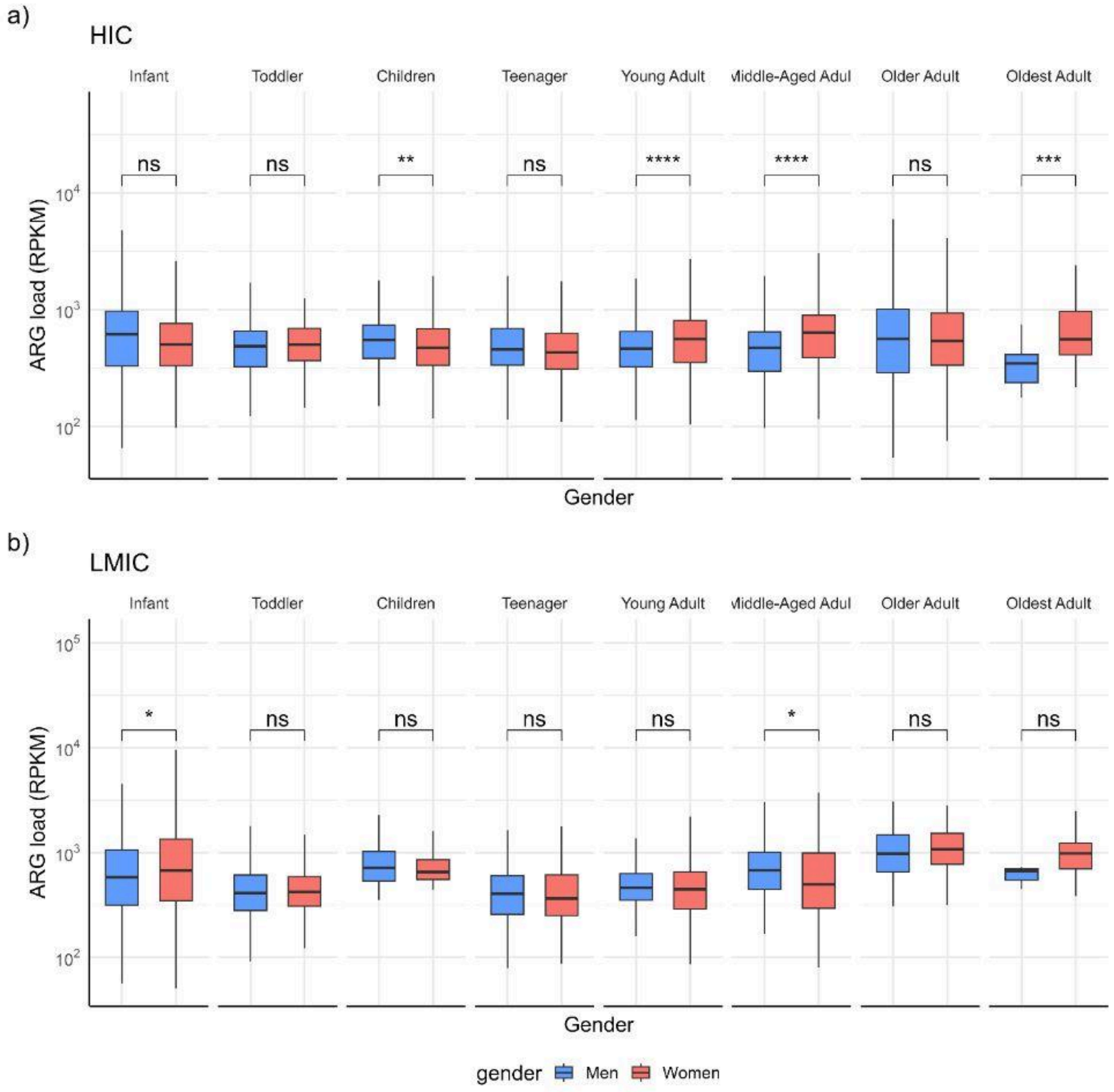

HIC

c)

Age Category	N (Women)	N (Men)	Effect Size (r)	Lower 95% CI	Upper 95% CI	Adjusted p-value
Infant	155	184	0.086	-0.020	0.192	0.1135
Toddler	96	98	0.064	-0.077	0.206	0.3700
Children	347	402	0.094	0.023	0.166	0.0097
Teenager	355	352	0.072	-0.002	0.146	0.0557
Young Adult	804	620	0.151	0.099	0.202	p<0.0001
Middle-Aged Adult	732	943	0.216	0.169	0.263	p<0.0001
Older Adult	150	224	0.009	-0.092	0.111	0.8579
Oldest Adult	30	52	0.406	0.206	0.606	0.0002

Supplementary Figure 6. Antibiotic resistance gene (ARG) load by gender and age group in **a)** high-income countries (HIC) and **b)** in low and middle-income countries (LMIC). The boxes display the median values and interquartile range (IQR) whiskers. For age category definitions, see *Methods*. Statistical significance is denoted as follows: **ns**: Not significant, $p > 0.05$. *: $p \leq 0.05$, **: $p \leq 0.01$, ***: $p \leq 0.001$,****: $p \leq 0.0001$. **c)** Gender differences in ARG load were tested using Wilcoxon test. Effect sizes (r) with 95% confidence intervals and sample sizes (N) are shown for high-income countries (HIC, c) and low- and middle-income countries (LMIC, d). Raw p-values were adjusted using Benjamini-Hochberg correction.

a)

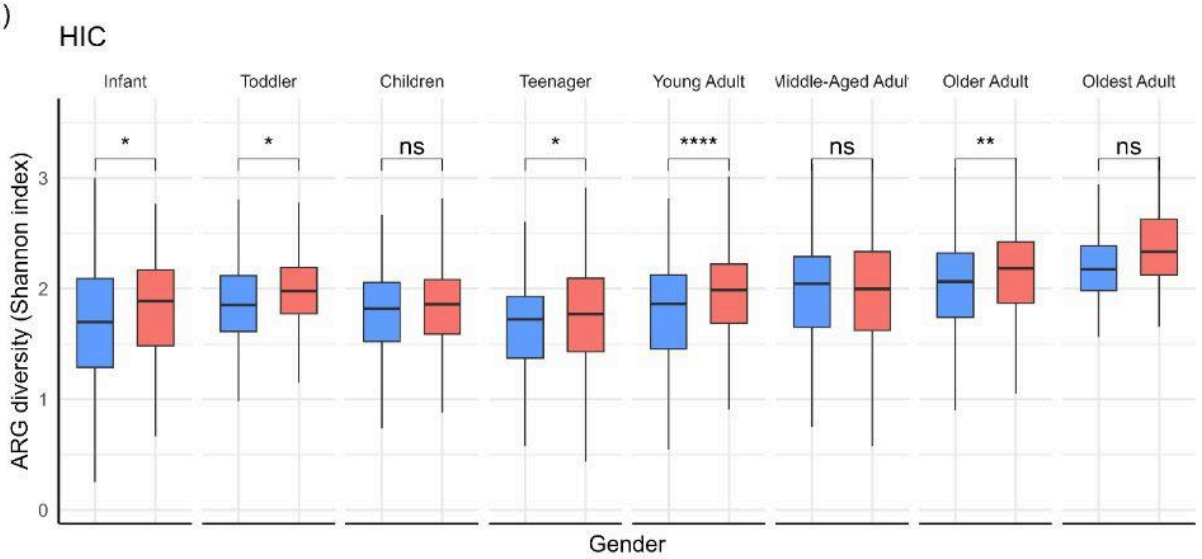

b)

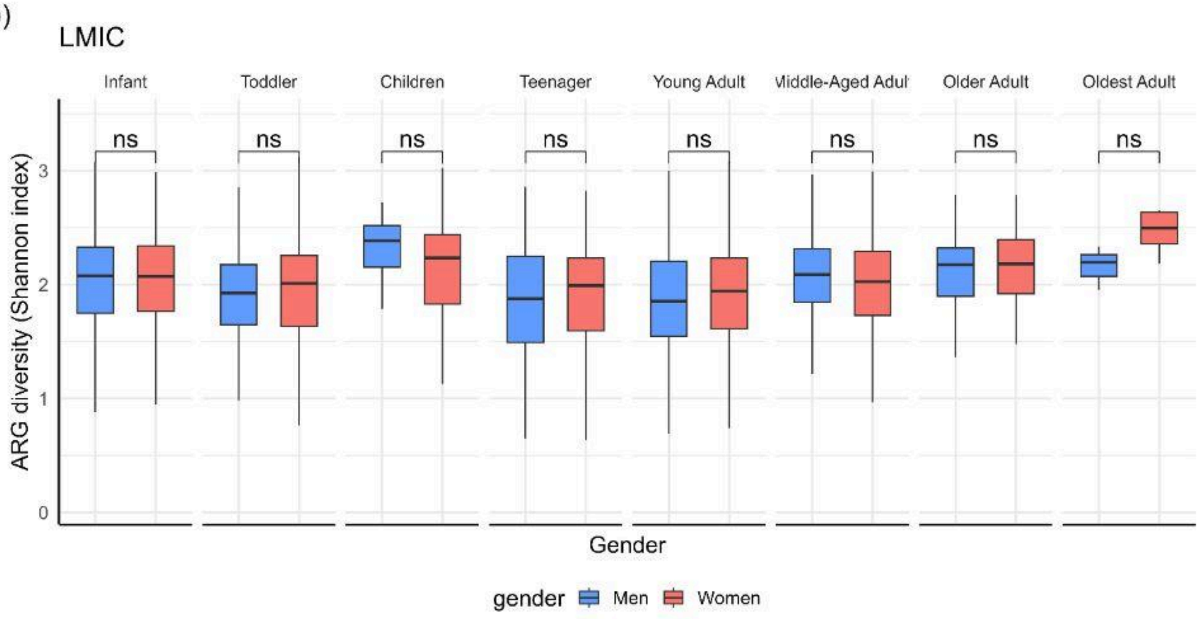

HIC

c)

Age Category	N (Women)	N (Men)	Effect Size (r)	Lower 95% CI	Upper 95% CI	Adjusted p-value
Infant	155	184	0.110	0.004	0.216	0.0433
Toddler	96	98	0.144	0.004	0.284	0.0448
Children	347	402	0.038	-0.034	0.110	0.2995
Teenager	355	352	0.094	0.021	0.168	0.0124
Young Adult	804	620	0.156	0.105	0.208	p<0.0001
Middle-Aged Adult	732	943	0.010	-0.037	0.058	0.6688
Older Adult	150	224	0.136	0.035	0.237	0.0085
Oldest Adult	30	52	0.199	-0.016	0.414	0.0726

Supplementary Figure 7. Age, gender, and antibiotic resistance gene diversity Gender-specific variations in ARG diversity across different age groups in **a)** high-income countries (HICs) and **b)** low- and middle-income countries (LMICs). Each box represents the median ARG diversity, with whiskers indicating the interquartile range (IQR). For age category definitions, see *Methods*. Statistical significance is denoted as follows: **ns**: Not significant, $p > 0.05$. *****: $p \leq 0.05$, ******: $p \leq 0.01$, *******: $p \leq 0.001$,********: $p \leq 0.0001$. **c)** Gender differences in ARG diversity (Shannon index) were tested using Wilcoxon test. Effect sizes (r) with 95% confidence intervals and sample sizes (N) are shown for high-income countries (HIC, c) and low- and middle-income countries (LMIC, d). Raw p-values were adjusted using Benjamini-Hochberg correction.

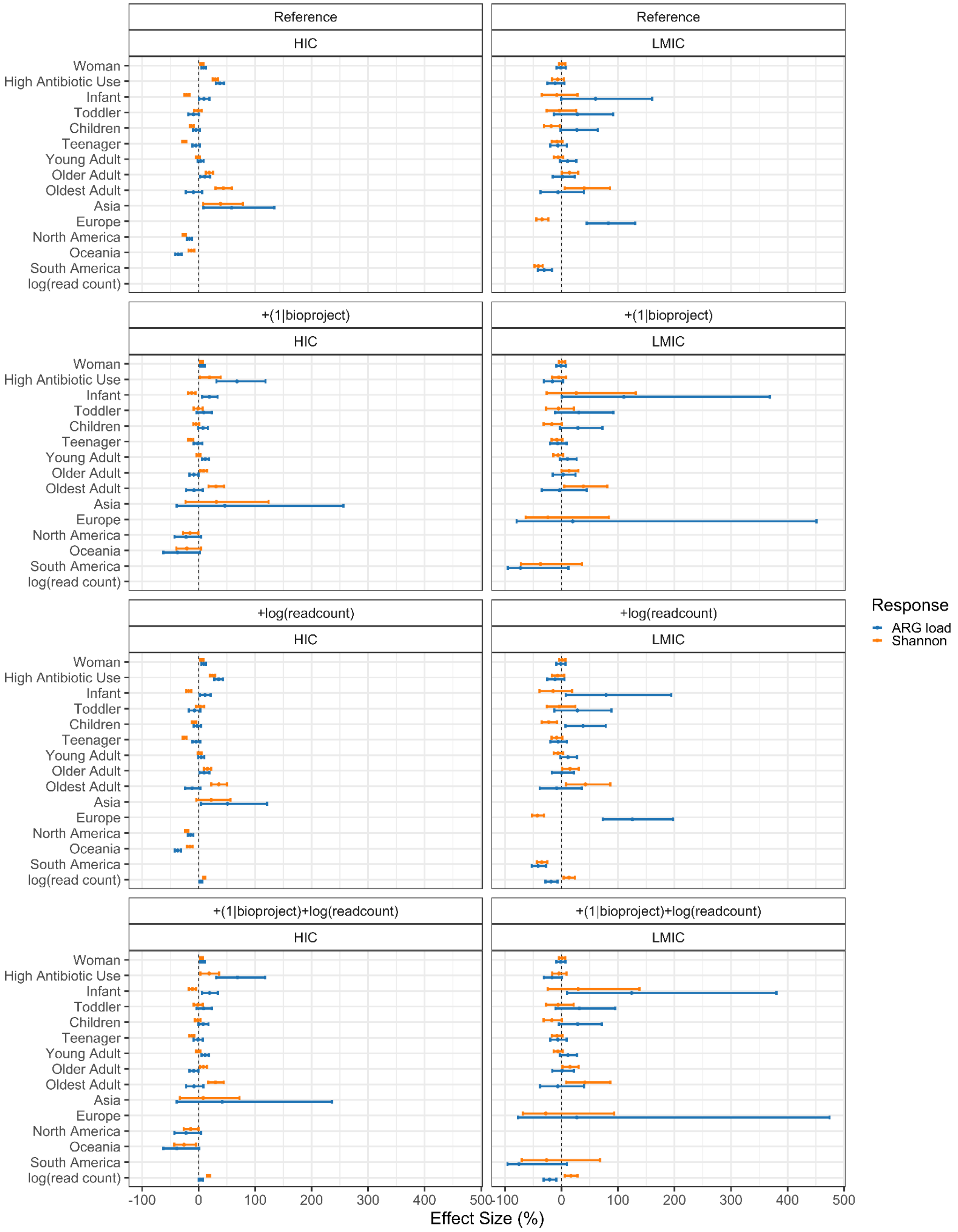

Reference
HIC
LMIC
+(1|bioproject)
+log(readcount)
+(1|bioproject)+log(readcount)
Woman
High Antibiotic Use
Infant
Toddler
Children
Teenager
Young Adult
Older Adult
Oldest Adult
Asia
Europe
North America
Oceania
South America
log(read count)
-100
0
100
200
300
400
500
Effect Size (%)
Response
ARG load
Shannon

Supplementary Figure 8. Comparison of drivers of Antibiotic resistance gene (ARG) load and diversity in high-income countries (HIC) and low- and middle-income countries (LMIC) across different linear models. Probabilistic 95% credible intervals for the effect sizes of socio-economic variables on ARG load (blue, modeled using log-normal regression), and diversity (orange, standard linear regression; see Supplementary Table 12). The *Reference* model refers to the primary model used in the main text, with covariates listed on the y-axis. The label *+(1|bioproject)* indicates a version of the reference model that includes a random intercept for study accession number, while *+log(readcount)* represents a model incorporating read count as model covariate. The baseline categories for region are Europe (for HIC) and Asia (for LMIC), and middle-aged adults for age. The effect sizes were mapped to percentage changes for easier interpretation using the formula 100×(exp(x) - 1).

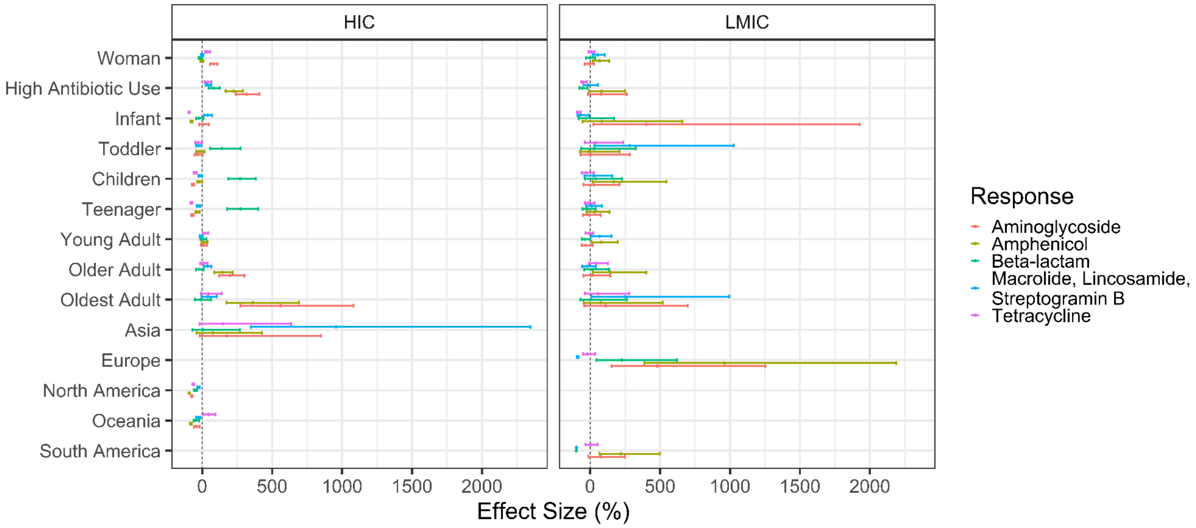

Supplementary Figure 9. Drivers of antibiotic-specific Antibiotic resistance gene (ARG) load in high-income countries (HIC) and low- and middle-income countries (LMIC). Probabilistic 95% credible intervals (CI) for the effect size of socio-economic variables on antibiotic-specific ARG load in a log-normal model (see Tables S13). The five most prevalent antibiotic resistance classes were selected. The baseline categories for region are Europe and Asia in HIC and LMIC, respectively, and middle-aged adults for age. The effect sizes were mapped to percentage changes for easier interpretation, using the transform 100×(exp(x) - 1).

Supplementary Table 1. Association between resistome composition and key covariates (PERMANOVA).

**Predictor**	**Df**	**Sum of Squares**	**R-squared**	**F-value**	**p-value**
Gender	1	5.68	0.0028	1.98e+01	<0.001 ***
Age category	5	52.82	0.028	3.68e+01	<0.001 ***
Region	4	99.78	0.049	8.68e+01	<0.001 ***
GDP per capita	1	2.61	0.0013	8.68e+01	<0.001 ***
Antibiotic use (DDD/1000 inhabitants)	1	10.8	0.0053	3.76e+01	<0.001 ***
Residual	6570	18884.26	0.92		
**Total**	6571	2055.95	1		
**Signif. codes:**					*** p < 0.001

Supplementary Table 2. Pairwise gender comparisons of ARG load within each age category in HICs and LMICs (Wilcoxon test).

Sample sizes are given in columns N1 and N2 for Group 1 and 2, respectively. Statistical significance is denoted as follows: **ns**: Not significant, p > 0.05. *: p ≤ 0.05, **: p ≤ 0.01, ***: p ≤ 0.001,****: p ≤ 0.0001.

Age	Group 1	Group 2	N1	N2	Group1 median	Group median	P_value	P.adj	P.adj significance	Effect direction
Infant	Women	Men	625	751	6.47	6.37	0.35	0.40	ns	Women > Men
Toddler	Women	Men	375	417	6.1	6.07	0.35	0.40	ns	Women > Men
Children	Women	Men	407	462	6.22	6.33	0.011	0.021	*	Men > Women
Teenager	Women	Men	473	430	6.07	6.11	0.11	0.18	ns	Men > Women
Young Adult	Women	Men	1139	844	6.29	6.17	7.51e-05	0.00025	***	Women > Men
Middle-Aged Adult	Women	Men	915	1126	6.44	6.21	1.01e-12	8.09e-12	****	Women > Men
Older Adult	Women	Men	206	319	6.57	6.47	0.581	0.58	ns	Women > Men
Oldest Adult	Women	Men	43	58	6.39	5.93	9.44e-05	0.00025	***	Women > Men

Supplementary Table 3. Pairwise gender comparisons of ARG diversity within each age category in HICs and LMICs (Wilcoxon test).

Sample sizes are given in columns N1 and N2 for Group 1 and 2, respectively. Statistical significance is denoted as follows: **ns**: Not significant, p > 0.05. *: p ≤ 0.05, **: p ≤ 0.01, ***: p ≤ 0.001,****: p ≤ 0.0001.

Age	Group 1	Group 2	N1	N2	Group median	Group median	P_value	P.adj	P.adj significance	Effect direction
Infant	Women	Men	625	751	2.03	2.02	0.184	0.25	ns	Women > Men
Toddler	Women	Men	375	417	2.01	1.92	0.037	0.059	ns	Women > Men
Children	Women	Men	407	462	1.92	1.87	0.436	0.50	ns	Women > Men
Teenager	Women	Men	473	430	1.84	1.75	0.0014	0.0057	**	Women > Men
Young Adult	Women	Men	1139	844	1.99	1.89	2.15e-08	1.72e-07	****	Women > Men
Middle-Aged Adult	Women	Men	915	1126	2.02	2.05	0.50	0.50	ns	Men > Women
Older Adult	Women	Men	206	319	2.18	2.1	0.023	0.059	ns	Women > Men
Oldest Adult	Women	Men	43	58	2.36	2.18	0.034	0.059	ns	Women > Men

Supplementary Table 4. Probabilistic log-normal linear regression for ARG load. The effect sizes and credible intervals were estimated using probabilistic multivariable linear regression. The effect estimate (posterior mean) is in relation to the baseline category (gender: man; age category: middle-aged adult; region: Asia in LMIC and Europe in HIC, GDP per capita: LMIC; antibiotics usage: low, <10). Q2.5 and Q97.5 denote the corresponding posterior percentiles and determine the probabilistic 95% credible intervals.

Predictor	Estimate	Q2.5	Q97.5	exp(Estimate)	exp(Q2.5)	exp(Q97.5)	Income Group
Intercept	6.388	6.19	6.589	594.59	487.746	727.306	LMIC
Woman	-0.008	-0.092	0.074	0.992	0.912	1.077	LMIC
Europe	0.604	0.369	0.834	1.829	1.446	2.302	LMIC
South America	-0.366	-0.544	-0.185	0.694	0.581	0.831	LMIC
Infant	0.473	-0.005	0.956	1.605	0.995	2.602	LMIC
Toddler	0.248	-0.146	0.649	1.281	0.864	1.914	LMIC
Children	0.239	-0.016	0.496	1.271	0.984	1.643	LMIC
Teenager	-0.065	-0.223	0.091	0.937	0.8	1.095	LMIC
Young Adult	0.103	-0.028	0.233	1.108	0.972	1.263	LMIC
Older Adult	0.019	-0.167	0.211	1.019	0.847	1.235	LMIC
Oldest Adult	-0.062	-0.466	0.334	0.94	0.627	1.397	LMIC
High Antibiotic Use	-0.118	-0.286	0.053	0.889	0.751	1.055	LMIC
Intercept	6.097	6.049	6.144	444.548	423.824	465.972	HIC
Woman	0.085	0.046	0.123	1.088	1.047	1.131	HIC
Asia	0.46	0.082	0.851	1.585	1.085	2.341	HIC
North America	-0.18	-0.231	-0.128	0.836	0.794	0.88	HIC
Oceania	-0.449	-0.537	-0.361	0.638	0.585	0.697	HIC
Infant	0.09	0.006	0.176	1.095	1.006	1.192	HIC
Toddler	-0.097	-0.2	0.006	0.908	0.819	1.006	HIC
Children	-0.044	-0.105	0.02	0.957	0.9	1.20	HIC
Teenager	-0.048	-0.117	0.022	0.953	0.889	1.022	HIC
Young Adult	0.033	-0.019	0.085	1.034	0.981	1.089	HIC
Older Adult	0.104	0.026	0.184	1.110	1.027	1.202	HIC
Oldest Adult	-0.097	-0.255	0.061	0.908	0.775	1.063	HIC
High Antibiotic Use	0.319	0.266	0.373	1.376	1.304	1.451	HIC

Supplementary Table 5. Probabilistic linear regression for ARG diversity. The effect sizes and credible intervals were estimated using probabilistic linear regression. The effect estimate (posterior mean) is in relation to the baseline category (gender: man; age category: middle-aged adult; region: Asia in LMIC, Europe in HIC, GDP per capita: LMIC; antibiotics usage: low, <10). Q2.5 and Q97.5 denote the corresponding posterior percentiles and determine the probabilistic 95% credible intervals.

Predictor	Estimate	Q2.5	Q97.5	exp(Estimate)	exp(Q2.5)	exp(Q97.5)	Income Group
Intercept	2.459	2.325	2.592	11.697	10.226	13.357	LMIC
Woman	0.011	-0.045	0.065	1.011	0.956	1.067	LMIC
Europe	-0.425	-0.588	-0.266	0.654	0.555	0.767	LMIC
South America	-0.529	-0.654	-0.403	0.589	0.52	0.668	LMIC
Infant	-0.087	-0.425	0.25	0.917	0.654	1.284	LMIC
Toddler	-0.034	-0.302	0.231	0.967	0.739	1.259	LMIC
Children	-0.201	-0.372	-0.031	0.818	0.689	0.97	LMIC
Teenager	-0.086	-0.19	0.019	0.917	0.827	1.019	LMIC
Young Adult	-0.059	-0.148	0.031	0.943	0.863	1.032	LMIC
Older Adult	0.134	0.005	0.26	1.143	1.005	1.297	LMIC
Oldest Adult	0.337	0.055	0.62	1.40	1.056	1.859	LMIC
High Antibiotic Use	-0.071	-0.181	0.041	0.932	0.835	1.042	LMIC
Intercept	1.907	1.875	1.939	6.735	6.523	6.95	HIC
Woman	0.057	0.032	0.082	1.059	1.033	1.085	HIC
Asia	0.328	0.076	0.579	1.388	1.079	1.785	HIC
North America	-0.292	-0.327	-0.256	0.747	0.721	0.774	HIC
Oceania	-0.138	-0.197	-0.078	0.871	0.821	0.925	HIC
Infant	-0.235	-0.289	-0.177	0.791	0.749	0.838	HIC
Toddler	-0.014	-0.084	0.054	0.986	0.92	1.056	HIC
Children	-0.128	-0.169	-0.085	0.88	0.844	0.918	HIC
Teenager	-0.293	-0.34	-0.247	0.746	0.712	0.781	HIC
Young Adult	-0.011	-0.045	0.025	0.989	0.956	1.025	HIC
Older Adult	0.172	0.12	0.226	1.188	1.127	1.254	HIC
Oldest Adult	0.363	0.262	0.463	1.438	1.299	1.589	HIC
High Antibiotic Use	0.258	0.222	0.293	1.294	1.249	1.34	HIC

Supplementary Table 6. Post-hoc test of ARG load (log RPKM) between regions (HICs and LMICs) (Tukey's post hoc test for multivariable regression). Sample sizes are given in columns N1 and N2 for Group 1 and 2, respectively. Statistical significance is denoted as follows: **ns**: Not significant, $p > 0.05$. *****: $p \le 0.05$, ******: $p \le 0.01$, ***: $p \le 0.001$,****: $p \le 0.0001$. The African region did not have enough adult samples to analyze.

Group 1	Group 2	N1	N2	Income Group	P.adj	P.adj Significant	Effect Direction
Europe	Asia	610	9	HIC	0.0107	*	Europe < Asia
North America	Asia	1775	9	HIC	0.00347	**	North America < Asia
Oceania	Asia	275	9	HIC	<0.001	***	Oceania < Asia
North America	Europe	1775	610	HIC	0.0523	ns	North America < Europe
Oceania	Europe	275	610	HIC	<0.001	****	Oceania < Europe
Oceania	North America	275	1775	HIC	<0.001	***	Oceania < North America
Europe	Asia	94	78	LMIC	<0.001	****	Europe > Asia
South America	Asia	443	78	LMIC	<0.001	***	South America < Asia
South America	Europe	443	94	LMIC	<0.001	****	South America < Europe

Supplementary Table 7. Post-hoc test of ARG diversity between regions (HICs and LMICs) (Tukey's post hoc test for multivariable regression).Sample sizes are given in columns N1 and N2 for Group 1 and 2, respectively. Statistical significance is denoted as follows: **ns**: Not significant, p > 0.05. *: p ≤ 0.05, **: p ≤ 0.01, ***: p ≤ 0.001,****: p ≤ 0.0001. The African region did not have enough adult samples to analyze.

**Group 1**	**Group 2**	**N1**	**N2**	**Income Group**	**P.adj**	**P.adj Significant**	**Effect Direction**
Europe	Asia	610	9	HIC	0.331	ns	Europe < Asia
North America	Asia	1775	9	HIC	<0.001	***	North America < Asia
Oceania	Asia	275	9	HIC	0.0788	ns	Oceania < Asia
North America	Europe	1775	610	HIC	<0.001	****	North America < Europe
Oceania	Europe	275	610	HIC	0.011	*	Oceania < Europe
Oceania	North America	275	1775	HIC	<0.001	****	Oceania > North America
Europe	Asia	94	78	LMIC	<0.001	***	Europe < Asia
South America	Asia	443	78	LMIC	<0.001	****	South America < Asia
South America	Europe	443	94	LMIC	0.383	ns	South America < Europe

Supplementary Table 8. Post-hoc test for ARG load (log RPKM) between age categories among (HICs and LMICs) (Tukey's post hoc test for multivariable regression). Sample sizes are given in columns N1 and N2 for Group 1 and 2, respectively. Statistical significance is denoted as follows: **ns**: Not significant, p > 0.05. *: p ≤ 0.05, **: p ≤ 0.01, ***: p ≤ 0.001,****: p ≤ 0.0001.

Group 1	Group 2	N1	N2	Income Group	P.adj	P.adj Significant	Effect Direction
Toddler	Infant	96	155	HIC	1	ns	Toddler < Infant
Children	Infant	347	155	HIC	1	ns	Children < Infant
Teenager	Infant	355	155	HIC	1	ns	Teenager < Infant
Young Adult	Infant	804	155	HIC	1	ns	Young Adult > Infant
Middle-Aged Adult	Infant	732	155	HIC	0.89	ns	Middle-Aged Adult > Infant
Older Adult	Infant	150	155	HIC	1	ns	Older Adult > Infant
Oldest Adult	Infant	30	155	HIC	1	ns	Oldest Adult > Infant
Children	Toddler	347	96	HIC	1	ns	Children < Toddler
Teenager	Toddler	355	96	HIC	1	ns	Teenager < Toddler
Young Adult	Toddler	804	96	HIC	1	ns	Young Adult > Toddler
Middle-Aged Adult	Toddler	732	96	HIC	0.784	ns	Middle-Aged Adult > Toddler
Older Adult	Toddler	150	96	HIC	1	ns	Older Adult > Toddler
Oldest Adult	Toddler	30	96	HIC	1	ns	Oldest Adult > Toddler
Teenager	Children	355	347	HIC	1	ns	Teenager < Children
Young Adult	Children	804	347	HIC	0.0232	*	Young Adult > Children
Middle-Aged Adult	Children	732	347	HIC	<0.001	***	Middle-Aged Adult > Children
Older Adult	Children	150	347	HIC	0.686	ns	Older Adult > Children
Oldest Adult	Children	30	347	HIC	0.89	ns	Oldest Adult > Children
Young Adult	Teenager	804	355	HIC	0.0894	ns	Young Adult > Teenager
Middle-Aged Adult	Teenager	732	355	HIC	0.00159	**	Middle-Aged Adult > Teenager
Older Adult	Teenager	150	355	HIC	0.784	ns	Older Adult > Teenager
Oldest Adult	Teenager	30	355	HIC	0.89	ns	Oldest Adult > Teenager
Middle-Aged Adult	Young Adult	732	804	HIC	1	ns	Middle-Aged Adult > Young Adult
Older Adult	Young Adult	150	804	HIC	1	ns	Older Adult > Young Adult

Oldest Adult	Young Adult	30	804	HIC	1	ns	Oldest Adult > Young Adult
Older Adult	Middle-Aged Adult	150	732	HIC	1	ns	Older Adult < Middle-Aged Adult
Oldest Adult	Middle-Aged Adult	30	732	HIC	1	ns	Oldest Adult > Middle-Aged Adult
Oldest Adult	Older Adult	30	150	HIC	1	ns	Oldest Adult > Older Adult
Toddler	Infant	7	6	LMIC	1	ns	Toddler < Infant
Children	Infant	28	6	LMIC	1	ns	Children < Infant
Teenager	Infant	97	6	LMIC	1	ns	Teenager < Infant
Young Adult	Infant	303	6	LMIC	1	ns	Young Adult < Infant
Middle-Aged Adult	Infant	121	6	LMIC	1	ns	Middle-Aged Adult < Infant
Older Adult	Infant	44	6	LMIC	1	ns	Older Adult < Infant
Oldest Adult	Infant	9	6	LMIC	1	ns	Oldest Adult < Infant
Children	Toddler	28	7	LMIC	1	ns	Children > Toddler
Teenager	Toddler	97	7	LMIC	1	ns	Teenager < Toddler
Young Adult	Toddler	303	7	LMIC	1	ns	Young Adult > Toddler
Middle-Aged Adult	Toddler	121	7	LMIC	1	ns	Middle-Aged Adult < Toddler
Older Adult	Toddler	44	7	LMIC	1	ns	Older Adult < Toddler
Oldest Adult	Toddler	9	7	LMIC	1	ns	Oldest Adult < Toddler
Teenager	Children	97	28	LMIC	1	ns	Teenager < Children
Young Adult	Children	303	28	LMIC	1	ns	Young Adult < Children
Middle-Aged Adult	Children	121	28	LMIC	1	ns	Middle-Aged Adult < Children
Older Adult	Children	44	28	LMIC	1	ns	Older Adult < Children
Oldest Adult	Children	9	28	LMIC	1	ns	Oldest Adult < Children
Young Adult	Teenager	303	97	LMIC	1	ns	Young Adult > Teenager
Middle-Aged Adult	Teenager	121	97	LMIC	1	ns	Middle-Aged Adult < Teenager
Older Adult	Teenager	44	97	LMIC	1	ns	Older Adult < Teenager
Oldest Adult	Teenager	9	97	LMIC	1	ns	Oldest Adult < Teenager
Middle-Aged Adult	Young Adult	121	303	LMIC	1	ns	Middle-Aged Adult < Young Adult
Older Adult	Young Adult	44	303	LMIC	1	ns	Older Adult < Young Adult

Oldest Adult	Young Adult	9	303	LMIC	1	ns	Oldest Adult < Young Adult
Older Adult	Middle-Aged Adult	44	121	LMIC	1	ns	Older Adult < Middle-Aged Adult
Oldest Adult	Middle-Aged Adult	9	121	LMIC	1	ns	Oldest Adult < Middle-Aged Adult
Oldest Adult	Older Adult	9	44	LMIC	1	ns	Oldest Adult > Older Adult

Supplementary Table 9. Post-hoc test for ARG diversity between age categories (LICs and LMICs) (Tukey's post hoc test for multivariable regression). Sample sizes are given in columns N1 and N2 for Group 1 and 2, respectively. Statistical significance is denoted as follows: **ns**: Not significant, p > 0.05. *: p ≤ 0.05, **: p ≤ 0.01, ***: p ≤ 0.001,****: p ≤ 0.0001.

Group 1	Group 2	N1	N2	Income Group	P.adj	P.adj Significant	Effect Direction
Toddler	Infant	96	155	HIC	0.0341	*	Toddler > Infant
Children	Infant	347	155	HIC	1	ns	Children > Infant
Teenager	Infant	355	155	HIC	0.191	ns	Teenager < Infant
Young Adult	Infant	804	155	HIC	<0.001	****	Young Adult > Infant
Middle-Aged Adult	Infant	732	155	HIC	0.0578	ns	Middle-Aged Adult > Infant
Older Adult	Infant	150	155	HIC	<0.001	****	Older Adult > Infant
Oldest Adult	Infant	30	155	HIC	<0.001	****	Oldest Adult > Infant
Children	Toddler	347	96	HIC	0.0989	ns	Children < Toddler
Teenager	Toddler	355	96	HIC	<0.001	****	Teenager < Toddler
Young Adult	Toddler	804	96	HIC	1	ns	Young Adult > Toddler
Middle-Aged Adult	Toddler	732	96	HIC	0.919	ns	Middle-Aged Adult < Toddler
Older Adult	Toddler	150	96	HIC	0.0465	*	Older Adult > Toddler
Oldest Adult	Toddler	30	96	HIC	0.00229	**	Oldest Adult > Toddler
Teenager	Children	355	347	HIC	0.00191	**	Teenager < Children
Young Adult	Children	804	347	HIC	<0.001	****	Young Adult > Children
Middle-Aged Adult	Children	732	347	HIC	0.149	ns	Middle-Aged Adult > Children
Older Adult	Children	150	347	HIC	<0.001	****	Older Adult > Children
Oldest Adult	Children	30	347	HIC	<0.001	****	Oldest Adult > Children
Young Adult	Teenager	804	355	HIC	<0.001	****	Young Adult > Teenager
Middle-Aged Adult	Teenager	732	355	HIC	<0.001	****	Middle-Aged Adult > Teenager
Older Adult	Teenager	150	355	HIC	<0.001	****	Older Adult > Teenager
Oldest Adult	Teenager	30	355	HIC	<0.001	****	Oldest Adult > Teenager
Middle-Aged Adult	Young Adult	732	804	HIC	0.0341	*	Middle-Aged Adult < Young Adult
Older Adult	Young Adult	150	804	HIC	<0.001	***	Older Adult > Young Adult

Oldest Adult	Young Adult	30	804	HIC	<0.001	***	Oldest Adult > Young Adult
Older Adult	Middle-Aged Adult	150	732	HIC	<0.001	****	Older Adult > Middle-Aged Adult
Oldest Adult	Middle-Aged Adult	30	732	HIC	<0.001	****	Oldest Adult > Middle-Aged Adult
Oldest Adult	Older Adult	30	150	HIC	0.391	ns	Oldest Adult > Older Adult
Toddler	Infant	7	6	LMIC	1	ns	Toddler > Infant
Children	Infant	28	6	LMIC	1	ns	Children < Infant
Teenager	Infant	97	6	LMIC	1	ns	Teenager > Infant
Young Adult	Infant	303	6	LMIC	1	ns	Young Adult > Infant
Middle-Aged Adult	Infant	121	6	LMIC	1	ns	Middle-Aged Adult > Infant
Older Adult	Infant	44	6	LMIC	1	ns	Older Adult > Infant
Oldest Adult	Infant	9	6	LMIC	1	ns	Oldest Adult > Infant
Children	Toddler	28	7	LMIC	1	ns	Children < Toddler
Teenager	Toddler	97	7	LMIC	1	ns	Teenager < Toddler
Young Adult	Toddler	303	7	LMIC	1	ns	Young Adult < Toddler
Middle-Aged Adult	Toddler	121	7	LMIC	1	ns	Middle-Aged Adult < Toddler
Older Adult	Toddler	44	7	LMIC	1	ns	Older Adult > Toddler
Oldest Adult	Toddler	9	7	LMIC	1	ns	Oldest Adult > Toddler
Teenager	Children	97	28	LMIC	1	ns	Teenager > Children
Young Adult	Children	303	28	LMIC	1	ns	Young Adult > Children
Middle-Aged Adult	Children	121	28	LMIC	1	ns	Middle-Aged Adult > Children
Older Adult	Children	44	28	LMIC	1	ns	Older Adult > Children
Oldest Adult	Children	9	28	LMIC	0.95	ns	Oldest Adult > Children
Young Adult	Teenager	303	97	LMIC	1	ns	Young Adult > Teenager
Middle-Aged Adult	Teenager	121	97	LMIC	1	ns	Middle-Aged Adult > Teenager
Older Adult	Teenager	44	97	LMIC	1	ns	Older Adult > Teenager
Oldest Adult	Teenager	9	97	LMIC	1	ns	Oldest Adult > Teenager
Middle-Aged Adult	Young Adult	121	303	LMIC	1	ns	Middle-Aged Adult > Young Adult
Older Adult	Young Adult	44	303	LMIC	1	ns	Older Adult > Young Adult

Oldest Adult	Young Adult	9	303	LMIC	1	ns	Oldest Adult > Young Adult
Older Adult	Middle-Aged Adult	44	121	LMIC	1	ns	Older Adult > Middle-Aged Adult
Oldest Adult	Middle-Aged Adult	9	121	LMIC	1	ns	Oldest Adult > Middle-Aged Adult
Oldest Adult	Older Adult	9	44	LMIC	1	ns	Oldest Adult > Older Adult

Supplementary Table 10. Variance inflation factor (VIF) for predictors. Variance Inflation Factor (VIF) analysis was conducted to assess multicollinearity among the predictors in the LMs. All VIF values remained below the commonly accepted threshold of 5, indicating that multicollinearity was not a significant concern in the models.

**Predictor**	**VIF**
Gender	1.017
Age category	1.051
Region	1.26
GDP per capita	1.75
Antibiotic use	1.58

Supplementary Table 11. Sample distribution by age and gender

	Infant	Toddler	Children	Teenager	Young adult	Middle-aged adult	Older adult	Oldest adult
**Women**	625	375	407	473	1139	915	206	43
**Men**	751	417	462	430	844	1126	319	58

Supplementary Table 12. Comparison of factors influencing ARG load and diversity in high-income countries (HIC) and low- and middle-income countries (LMIC) across different linear models. This table presents probabilistic effect size estimates of socio-economic variables on ARG load (modeled using log-normal regression) and Shannon diversity (modeled using standard linear regression). The *Model* specifies the model variant, with *Reference* referring to the primary model used in the main text. The notation *+(1|bioproject)* indicates the inclusion of a random intercept for study accession number, while *log(readcount)* denotes the addition of read count as a covariate. The posterior 2.5% and 97.5% quantiles are reported in the *Q2.5* and *Q97.5* columns, respectively, and the *Significant* column indicates whether this interval contains zero. The baseline categories for region are Europe (for HIC) and Asia (for LMIC), while middle-aged adults serve as the reference category for age. Effect sizes were transformed into percentage changes for easier interpretation using the formula 100×(exp(*x*) - 1).

**Predictor**	**Response**	**Income Group**	**Model**	**Estimate**	**Q2.5**	**Q97.5**	**exp(Est.)**	**exp(Q2.5)**	**exp(Q97.5)**	**Sig.**
Intercept	Shannon	HIC	Reference	1.907	1.875	1.939	6.735	6.523	**6.95**	*
Intercept	Shannon	HIC	+(1|bioproject)	1.905	1.749	2.052	6.72	5.749	**7.787**	*
Intercept	Shannon	HIC	+log(readcount)	1.741	1.698	1.784	5.702	5.462	**5.956**	*
**Intercept**	**Shannon**	**HIC**	**+(1|bioproject)+log(readcount)**	**1.633**	**1.479**	**1.78**	**5.118**	**4.387**	**5.931**	*
**Intercept**	**Shannon**	**LMIC**	**Reference**	**2.459**	**2.325**	**2.592**	**11.697**	**10.226**	**13.357**	*
**Intercept**	**Shannon**	**LMIC**	**+(1|bioproject)**	**2.286**	**1.651**	**2.678**	**9.835**	**5.211**	**14.561**	*
**Intercept**	**Shannon**	**LMIC**	**+log(readcount)**	**2.221**	**2.007**	**2.435**	**9.215**	**7.445**	**11.412**	*
**Intercept**	**Shannon**	**LMIC**	**+(1|bioproject)+log(readcount)**	**1.883**	**1.183**	**2.376**	**6.574**	**3.264**	**10.762**	*
**Intercept**	**ARG load**	**HIC**	**Reference**	**6.097**	**6.049**	**6.144**	**444.548**	**423.824**	**465.972**	*
**Intercept**	**ARG load**	**HIC**	**+(1|bioproject)**	**5.942**	**5.665**	**6.203**	**380.688**	**288.672**	**494.284**	*
**Intercept**	**ARG load**	**HIC**	**+log(readcount)**	**6.026**	**5.96**	**6.093**	**413.984**	**387.624**	**442.675**	*
**Intercept**	**ARG load**	**HIC**	**+(1|bioproject)+log(readcount)**	**5.873**	**5.593**	**6.138**	**355.345**	**268.668**	**462.934**	*
**Intercept**	**ARG load**	**LMIC**	**Reference**	**6.388**	**6.19**	**6.589**	**594.59**	**487.746**	**727.306**	*
**Intercept**	**ARG load**	**LMIC**	**+(1|bioproject)**	**5.074**	**2.384**	**6.64**	**159.889**	**10.85**	**765.174**	*
**Intercept**	**ARG load**	**LMIC**	**+log(readcount)**	**6.784**	**6.456**	**7.104**	**883.928**	**636.547**	**1216.387**	*

Intercept	ARG load	LMIC	+(1|bioproject)+ log(readcount)	5.482	2.588	7.188	240.266	13.301	1323.619	*
Woman	Shannon	HIC	Reference	0.057	0.032	0.082	1.059	1.033	1.085	*
Woman	Shannon	HIC	+(1|bioproject)	0.048	0.025	0.07	1.049	1.025	1.073	*
Woman	Shannon	HIC	+log(readcount)	0.055	0.031	0.08	1.057	1.031	1.083	*
Woman	Shannon	HIC	+(1|bioproject)+ log(readcount)	0.048	0.026	0.072	1.05	1.026	1.074	*
Woman	Shannon	LMIC	Reference	0.011	-0.045	0.065	1.011	0.956	1.067	ns
Woman	Shannon	LMIC	+(1|bioproject)	0.008	-0.046	0.064	1.008	0.955	1.066	ns
Woman	Shannon	LMIC	+log(readcount)	0.011	-0.044	0.066	1.012	0.956	1.068	ns
Woman	Shannon	LMIC	+(1|bioproject)+ log(readcount)	0.009	-0.046	0.064	1.009	0.955	1.066	ns
Woman	ARG load	HIC	Reference	0.085	0.046	0.123	1.088	1.047	1.131	*
Woman	ARG load	HIC	+(1|bioproject)	0.065	0.03	0.099	1.067	1.031	1.104	*
Woman	ARG load	HIC	+log(readcount)	0.084	0.046	0.121	1.087	1.047	1.129	*
Woman	ARG load	HIC	+(1|bioproject)+ log(readcount)	0.065	0.03	0.1	1.067	1.03	1.105	*
Woman	ARG load	LMIC	Reference	-0.008	-0.092	0.074	0.992	0.912	1.077	ns
Woman	ARG load	LMIC	+(1|bioproject)	-0.01	-0.096	0.074	0.99	0.909	1.077	ns
Woman	ARG load	LMIC	+log(readcount)	-0.012	-0.098	0.07	0.988	0.907	1.073	ns
Woman	ARG load	LMIC	+(1|bioproject)+ log(readcount)	-0.014	-0.096	0.069	0.986	0.909	1.072	ns
High Antibiotic Use	Shannon	HIC	Reference	0.258	0.222	0.293	1.294	1.249	1.34	*
High Antibiotic Use	Shannon	HIC	+(1|bioproject)	0.177	0.024	0.33	1.194	1.024	1.391	*

High Antibiotic Use	Shannon	HIC	+log(readcount)	0.221	0.185	0.258	1.248	1.204	1.294	*
High Antibiotic Use	Shannon	HIC	+(1|bioproject)+ log(readcount)	0.171	0.032	0.309	1.187	1.033	1.362	*
High Antibiotic Use	Shannon	LMIC	Reference	-0.071	-0.181	0.041	0.932	0.835	1.042	ns
High Antibiotic Use	Shannon	LMIC	+(1|bioproject)	-0.05	-0.18	0.078	0.951	0.835	1.082	ns
High Antibiotic Use	Shannon	LMIC	+log(readcount)	-0.07	-0.188	0.045	0.932	0.829	1.046	ns
High Antibiotic Use	Shannon	LMIC	+(1|bioproject)+ log(readcount)	-0.046	-0.178	0.085	0.955	0.837	1.089	ns
High Antibiotic Use	ARG load	HIC	Reference	0.319	0.266	0.373	1.376	1.304	1.451	*
High Antibiotic Use	ARG load	HIC	+(1|bioproject)	0.518	0.274	0.781	1.679	1.315	2.183	*
High Antibiotic Use	ARG load	HIC	+log(readcount)	0.303	0.246	0.359	1.354	1.279	1.432	*
High Antibiotic Use	ARG load	HIC	+(1|bioproject)+ log(readcount)	0.524	0.272	0.777	1.689	1.312	2.175	*

**High Antibiotic Use**	**ARG load**	**LMIC**	**Reference**	**-0.118**	**-0.286**	**0.053**	**0.889**	**0.751**	**1.055**	**ns**
**High Antibiotic Use**	**ARG load**	**LMIC**	**+(1|bioproject)**	**-0.174**	**-0.374**	**0.028**	**0.84**	**0.688**	**1.028**	**ns**
**High Antibiotic Use**	**ARG load**	**LMIC**	**+log(readcount)**	**-0.122**	**-0.292**	**0.052**	**0.886**	**0.747**	**1.053**	**ns**
**High Antibiotic Use**	**ARG load**	**LMIC**	**+(1|bioproject)+log(readcount)**	**-0.181**	**-0.374**	**0.011**	**0.834**	**0.688**	**1.011**	**ns**
**Infant**	**Shannon**	**HIC**	**Reference**	**-0.235**	**-0.289**	**-0.177**	**0.791**	**0.749**	**0.838**	*****
**Infant**	**Shannon**	**HIC**	**+(1|bioproject)**	**-0.131**	**-0.207**	**-0.055**	**0.877**	**0.813**	**0.947**	*****
**Infant**	**Shannon**	**HIC**	**+log(readcount)**	**-0.193**	**-0.249**	**-0.137**	**0.824**	**0.78**	**0.872**	*****
**Infant**	**Shannon**	**HIC**	**+(1|bioproject)+ log(readcount)**	**-0.117**	**-0.19**	**-0.044**	**0.89**	**0.827**	**0.957**	*****
**Infant**	**Shannon**	**LMIC**	**Reference**	**-0.087**	**-0.425**	**0.25**	**0.917**	**0.654**	**1.284**	**ns**
**Infant**	**Shannon**	**LMIC**	**+(1|bioproject)**	**0.233**	**-0.302**	**0.839**	**1.262**	**0.739**	**2.313**	**ns**
**Infant**	**Shannon**	**LMIC**	**+log(readcount)**	**-0.159**	**-0.492**	**0.174**	**0.853**	**0.611**	**1.189**	**ns**
**Infant**	**Shannon**	**LMIC**	**+(1|bioproject)+ log(readcount)**	**0.261**	**-0.28**	**0.866**	**1.298**	**0.756**	**2.378**	**ns**
**Infant**	**ARG load**	**HIC**	**Reference**	**0.09**	**0.006**	**0.176**	**1.095**	**1.006**	**1.192**	*****
**Infant**	**ARG load**	**HIC**	**+(1|bioproject)**	**0.174**	**0.059**	**0.291**	**1.191**	**1.06**	**1.337**	*****
**Infant**	**ARG load**	**HIC**	**+log(readcount)**	**0.108**	**0.022**	**0.192**	**1.114**	**1.022**	**1.212**	*****
**Infant**	**ARG load**	**HIC**	**+(1|bioproject)+ log(readcount)**	**0.177**	**0.057**	**0.294**	**1.193**	**1.059**	**1.342**	*****
**Infant**	**ARG load**	**LMIC**	**Reference**	**0.473**	**-0.005**	**0.956**	**1.605**	**0.995**	**2.602**	**ns**
**Infant**	**ARG load**	**LMIC**	**+(1|bioproject)**	**0.743**	**0.007**	**1.545**	**2.103**	**1.007**	**4.688**	*****

Infant	ARG load	LMIC	+log(readcount)	0.581	0.073	1.08	1.788	1.076	2.943	*
Infant	ARG load	LMIC	+(1|bioproject)+ log(readcount)	0.807	0.094	1.569	2.241	1.098	4.803	*
Toddler	Shannon	HIC	Reference	-0.014	-0.084	0.054	0.986	0.92	1.056	ns
Toddler	Shannon	HIC	+(1|bioproject)	-0.011	-0.094	0.068	0.989	0.91	1.071	ns
Toddler	Shannon	HIC	+log(readcount)	0.027	-0.044	0.096	1.027	0.957	1.101	ns
Toddler	Shannon	HIC	+(1|bioproject)+ log(readcount)	-0.011	-0.091	0.07	0.989	0.913	1.072	ns
Toddler	Shannon	LMIC	Reference	-0.034	-0.302	0.231	0.967	0.739	1.259	ns
Toddler	Shannon	LMIC	+(1|bioproject)	-0.053	-0.321	0.2	0.948	0.725	1.221	ns
Toddler	Shannon	LMIC	+log(readcount)	-0.039	-0.3	0.221	0.962	0.741	1.247	ns
Toddler	Shannon	LMIC	+(1|bioproject)+ log(readcount)	-0.061	-0.318	0.196	0.941	0.728	1.216	ns
Toddler	ARG load	HIC	Reference	-0.097	-0.2	0.006	0.908	0.819	1.006	ns
Toddler	ARG load	HIC	+(1|bioproject)	0.086	-0.038	0.209	1.09	0.963	1.233	ns
Toddler	ARG load	HIC	+log(readcount)	-0.078	-0.188	0.028	0.925	0.829	1.029	ns
Toddler	ARG load	HIC	+(1|bioproject)+ log(readcount)	0.086	-0.039	0.209	1.09	0.961	1.233	ns
Toddler	ARG load	LMIC	Reference	0.248	-0.146	0.649	1.281	0.864	1.914	ns
Toddler	ARG load	LMIC	+(1|bioproject)	0.268	-0.118	0.651	1.307	0.889	1.917	ns
Toddler	ARG load	LMIC	+log(readcount)	0.249	-0.135	0.633	1.283	0.874	1.883	ns
Toddler	ARG load	LMIC	+(1|bioproject)+ log(readcount)	0.274	-0.113	0.667	1.315	0.894	1.948	ns
Children	Shannon	HIC	Reference	-0.128	-0.169	-0.085	0.88	0.844	0.918	*
Children	Shannon	HIC	+(1|bioproject)	-0.047	-0.099	0.007	0.954	0.906	1.007	ns
Children	Shannon	HIC	+log(readcount)	-0.082	-0.125	-0.039	0.921	0.883	0.961	*
Children	Shannon	HIC	+(1|bioproject)+ log(readcount)	-0.019	-0.071	0.033	0.981	0.931	1.034	ns

**Children**	**Shannon**	**LMIC**	**Reference**	**-0.201**	**-0.372**	**-0.031**	**0.818**	**0.689**	**0.97**	*****
**Children**	**Shannon**	**LMIC**	**+(1|bioproject)**	**-0.187**	**-0.383**	**0.004**	**0.829**	**0.682**	**1.004**	**ns**
**Children**	**Shannon**	**LMIC**	**+log(readcount)**	**-0.256**	**-0.432**	**-0.081**	**0.774**	**0.649**	**0.922**	*****
**Children**	**Shannon**	**LMIC**	**+(1|bioproject)+ log(readcount)**	**-0.185**	**-0.377**	**0.008**	**0.831**	**0.686**	**1.008**	**ns**
**Children**	**ARG load**	**HIC**	**Reference**	**-0.044**	**-0.105**	**0.02**	**0.957**	**0.9**	**1.02**	**ns**
**Children**	**ARG load**	**HIC**	**+(1|bioproject)**	**0.073**	**-0.008**	**0.153**	**1.076**	**0.992**	**1.165**	**ns**
**Children**	**ARG load**	**HIC**	**+log(readcount)**	**-0.023**	**-0.089**	**0.042**	**0.977**	**0.915**	**1.043**	**ns**
**Children**	**ARG load**	**HIC**	**+(1|bioproject)+ log(readcount)**	**0.078**	**-0.004**	**0.161**	**1.081**	**0.996**	**1.174**	**ns**
**Children**	**ARG load**	**LMIC**	**Reference**	**0.239**	**-0.016**	**0.496**	**1.271**	**0.984**	**1.643**	**ns**
**Children**	**ARG load**	**LMIC**	**+(1|bioproject)**	**0.257**	**-0.026**	**0.544**	**1.293**	**0.974**	**1.723**	**ns**
**Children**	**ARG load**	**LMIC**	**+log(readcount)**	**0.323**	**0.066**	**0.579**	**1.381**	**1.068**	**1.784**	*****
**Children**	**ARG load**	**LMIC**	**+(1|bioproject)+ log(readcount)**	**0.252**	**-0.046**	**0.538**	**1.287**	**0.955**	**1.713**	**ns**
**Teenager**	**Shannon**	**HIC**	**Reference**	**-0.293**	**-0.34**	**-0.247**	**0.746**	**0.712**	**0.781**	*****
**Teenager**	**Shannon**	**HIC**	**+(1|bioproject)**	**-0.151**	**-0.203**	**-0.098**	**0.86**	**0.817**	**0.907**	*****
**Teenager**	**Shannon**	**HIC**	**+log(readcount)**	**-0.283**	**-0.33**	**-0.239**	**0.753**	**0.719**	**0.788**	*****
**Teenager**	**Shannon**	**HIC**	**+(1|bioproject)+ log(readcount)**	**-0.133**	**-0.183**	**-0.083**	**0.876**	**0.833**	**0.921**	*****
**Teenager**	**Shannon**	**LMIC**	**Reference**	**-0.086**	**-0.19**	**0.019**	**0.917**	**0.827**	**1.019**	**ns**
**Teenager**	**Shannon**	**LMIC**	**+(1|bioproject)**	**-0.086**	**-0.192**	**0.016**	**0.917**	**0.826**	**1.017**	**ns**
**Teenager**	**Shannon**	**LMIC**	**+log(readcount)**	**-0.09**	**-0.192**	**0.014**	**0.914**	**0.825**	**1.014**	**ns**
**Teenager**	**Shannon**	**LMIC**	**+(1|bioproject)+ log(readcount)**	**-0.084**	**-0.189**	**0.016**	**0.919**	**0.828**	**1.016**	**ns**
**Teenager**	**ARG load**	**HIC**	**Reference**	**-0.048**	**-0.117**	**0.022**	**0.953**	**0.889**	**1.022**	**ns**
**Teenager**	**ARG load**	**HIC**	**+(1|bioproject)**	**-0.015**	**-0.094**	**0.065**	**0.985**	**0.91**	**1.067**	**ns**
**Teenager**	**ARG load**	**HIC**	**+log(readcount)**	**-0.044**	**-0.115**	**0.027**	**0.957**	**0.892**	**1.028**	**ns**

Teenager	ARG load	HIC	+(1|bioproject)+ log(readcount)	-0.012	-0.095	0.073	0.988	0.91	1.075	ns
Teenager	ARG load	LMIC	Reference	-0.065	-0.223	0.091	0.937	0.8	1.095	ns
Teenager	ARG load	LMIC	+(1|bioproject)	-0.068	-0.227	0.088	0.934	0.797	1.092	ns
Teenager	ARG load	LMIC	+log(readcount)	-0.06	-0.218	0.091	0.942	0.804	1.096	ns
Teenager	ARG load	LMIC	+(1|bioproject)+ log(readcount)	-0.065	-0.219	0.089	0.937	0.803	1.094	ns
Young Adult	Shannon	HIC	Reference	-0.011	-0.045	0.025	0.989	0.956	1.025	ns
Young Adult	Shannon	HIC	+(1|bioproject)	-0.003	-0.04	0.032	0.997	0.961	1.033	ns
Young Adult	Shannon	HIC	+log(readcount)	0.017	-0.018	0.052	1.017	0.982	1.053	ns
Young Adult	Shannon	HIC	+(1|bioproject)+ log(readcount)	-0.008	-0.044	0.028	0.992	0.957	1.028	ns
Young Adult	Shannon	LMIC	Reference	-0.059	-0.148	0.031	0.943	0.863	1.032	ns
Young Adult	Shannon	LMIC	+(1|bioproject)	-0.062	-0.153	0.029	0.94	0.858	1.03	ns
Young Adult	Shannon	LMIC	+log(readcount)	-0.064	-0.15	0.025	0.938	0.861	1.025	ns
Young Adult	Shannon	LMIC	+(1|bioproject)+ log(readcount)	-0.064	-0.151	0.023	0.938	0.86	1.024	ns
Young Adult	ARG load	HIC	Reference	0.033	-0.019	0.085	1.034	0.981	1.089	ns
Young Adult	ARG load	HIC	+(1|bioproject)	0.113	0.058	0.17	1.12	1.06	1.185	*
Young Adult	ARG load	HIC	+log(readcount)	0.045	-0.008	0.096	1.046	0.992	1.101	ns

Young Adult	ARG load	HIC	+(1|bioproject)+ log(readcount)	0.111	0.052	0.167	1.117	1.054	1.182	*
Young Adult	ARG load	LMIC	Reference	0.103	-0.028	0.233	1.108	0.972	1.263	ns
Young Adult	ARG load	LMIC	+(1|bioproject)	0.101	-0.031	0.238	1.107	0.97	1.268	ns
Young Adult	ARG load	LMIC	+log(readcount)	0.108	-0.022	0.241	1.114	0.978	1.273	ns
Young Adult	ARG load	LMIC	+(1|bioproject)+ log(readcount)	0.109	-0.027	0.241	1.115	0.973	1.272	ns
Older Adult	Shannon	HIC	Reference	0.172	0.12	0.226	1.188	1.127	1.254	*
Older Adult	Shannon	HIC	+(1|bioproject)	0.085	0.031	0.14	1.089	1.031	1.15	*
Older Adult	Shannon	HIC	+log(readcount)	0.146	0.092	0.199	1.157	1.097	1.221	*
Older Adult	Shannon	HIC	+(1|bioproject)+ log(readcount)	0.08	0.024	0.136	1.084	1.024	1.145	*
Older Adult	Shannon	LMIC	Reference	0.134	0.005	0.26	1.143	1.005	1.297	*
Older Adult	Shannon	LMIC	+(1|bioproject)	0.128	-0.001	0.262	1.136	0.999	1.299	ns
Older Adult	Shannon	LMIC	+log(readcount)	0.141	0.012	0.268	1.151	1.012	1.307	*
Older Adult	Shannon	LMIC	+(1|bioproject)+ log(readcount)	0.141	0.016	0.265	1.151	1.016	1.303	*
Older Adult	ARG load	HIC	Reference	0.104	0.026	0.184	1.11	1.027	1.202	*
Older Adult	ARG load	HIC	+(1|bioproject)	-0.09	-0.176	-0.004	0.914	0.839	0.996	*

Older Adult	ARG load	HIC	+log(readcount)	0.093	0.015	0.173	1.097	1.015	1.189	*
Older Adult	ARG load	HIC	+(1|bioproject)+ log(readcount)	-0.091	-0.179	-0.004	0.913	0.836	0.996	*
Older Adult	ARG load	LMIC	Reference	0.019	-0.167	0.211	1.019	0.847	1.235	ns
Older Adult	ARG load	LMIC	+(1|bioproject)	0.027	-0.167	0.222	1.028	0.846	1.249	ns
Older Adult	ARG load	LMIC	+log(readcount)	0.001	-0.187	0.199	1.001	0.829	1.22	ns
Older Adult	ARG load	LMIC	+(1|bioproject)+ log(readcount)	0.009	-0.177	0.199	1.009	0.838	1.22	ns
Oldest Adult	Shannon	HIC	Reference	0.363	0.262	0.463	1.438	1.299	1.589	*
Oldest Adult	Shannon	HIC	+(1|bioproject)	0.269	0.161	0.373	1.308	1.175	1.452	*
Oldest Adult	Shannon	HIC	+log(readcount)	0.305	0.202	0.408	1.356	1.224	1.503	*
Oldest Adult	Shannon	HIC	+(1|bioproject)+ log(readcount)	0.262	0.157	0.368	1.3	1.169	1.445	*
Oldest Adult	Shannon	LMIC	Reference	0.337	0.055	0.62	1.4	1.056	1.859	*
Oldest Adult	Shannon	LMIC	+(1|bioproject)	0.326	0.047	0.594	1.385	1.048	1.812	*
Oldest Adult	Shannon	LMIC	+log(readcount)	0.353	0.078	0.623	1.424	1.082	1.864	*
Oldest Adult	Shannon	LMIC	+(1|bioproject)+ log(readcount)	0.347	0.081	0.622	1.415	1.084	1.863	*
Oldest Adult	ARG load	HIC	Reference	-0.097	-0.255	0.061	0.908	0.775	1.063	ns

Oldest Adult	ARG load	HIC	+(1|bioproject)	-0.086	-0.251	0.073	0.918	0.778	1.075	ns
Oldest Adult	ARG load	HIC	+log(readcount)	-0.123	-0.276	0.033	0.884	0.759	1.034	ns
Oldest Adult	ARG load	HIC	+(1|bioproject)+ log(readcount)	-0.086	-0.251	0.08	0.918	0.778	1.083	ns
Oldest Adult	ARG load	LMIC	Reference	-0.062	-0.466	0.334	0.94	0.627	1.397	ns
Oldest Adult	ARG load	LMIC	+(1|bioproject)	-0.033	-0.428	0.371	0.967	0.652	1.449	ns
Oldest Adult	ARG load	LMIC	+log(readcount)	-0.088	-0.487	0.306	0.916	0.614	1.358	ns
Oldest Adult	ARG load	LMIC	+(1|bioproject)+ log(readcount)	-0.067	-0.476	0.336	0.935	0.621	1.399	ns
Asia	Shannon	HIC	Reference	0.328	0.076	0.579	1.388	1.079	1.785	*
Asia	Shannon	HIC	+(1|bioproject)	0.273	-0.264	0.806	1.313	0.768	2.238	ns
Asia	Shannon	HIC	+log(readcount)	0.202	-0.042	0.447	1.224	0.959	1.564	ns
Asia	Shannon	HIC	+(1|bioproject)+ log(readcount)	0.079	-0.402	0.545	1.082	0.669	1.724	ns
Asia	ARG load	HIC	Reference	0.46	0.082	0.851	1.585	1.085	2.341	*
Asia	ARG load	HIC	+(1|bioproject)	0.381	-0.49	1.27	1.464	0.613	3.561	ns
Asia	ARG load	HIC	+log(readcount)	0.413	0.038	0.793	1.511	1.039	2.21	*
Asia	ARG load	HIC	+(1|bioproject)+ log(readcount)	0.35	-0.49	1.212	1.42	0.613	3.361	ns
Europe	Shannon	LMIC	Reference	-0.425	-0.588	-0.266	0.654	0.555	0.767	*
Europe	Shannon	LMIC	+(1|bioproject)	-0.278	-1.005	0.609	0.757	0.366	1.838	ns
Europe	Shannon	LMIC	+log(readcount)	-0.555	-0.741	-0.371	0.574	0.477	0.69	*
Europe	Shannon	LMIC	+(1|bioproject)+l og(readcount)	-0.322	-1.153	0.659	0.724	0.316	1.933	ns

Europe	ARG load	LMIC	Reference	0.604	0.369	0.834	1.829	1.446	2.302	*
Europe	ARG load	LMIC	+(1|bioproject)	0.181	-1.583	1.707	1.199	0.205	5.511	ns
Europe	ARG load	LMIC	+log(readcount)	0.813	0.549	1.09	2.255	1.731	2.976	*
Europe	ARG load	LMIC	+(1|bioproject)+ log(readcount)	0.24	-1.472	1.748	1.272	0.23	5.744	ns
North America	Shannon	HIC	Reference	-0.292	-0.327	-0.256	0.747	0.721	0.774	*
North America	Shannon	HIC	+(1|bioproject)	-0.165	-0.323	-0.005	0.848	0.724	0.995	*
North America	Shannon	HIC	+log(readcount)	-0.235	-0.271	-0.199	0.791	0.762	0.82	*
North America	Shannon	HIC	+(1|bioproject)+ log(readcount)	-0.154	-0.308	-0.002	0.857	0.735	0.998	*
North America	ARG load	HIC	Reference	-0.18	-0.231	-0.128	0.836	0.794	0.88	*
North America	ARG load	HIC	+(1|bioproject)	-0.251	-0.556	0.044	0.778	0.574	1.045	ns
North America	ARG load	HIC	+log(readcount)	-0.155	-0.212	-0.099	0.856	0.809	0.906	*
North America	ARG load	HIC	+(1|bioproject)+log(readcount)	-0.254	-0.558	0.04	0.776	0.572	1.041	ns
Oceania	Shannon	HIC	Reference	-0.138	-0.197	-0.078	0.871	0.821	0.925	*
Oceania	Shannon	HIC	+(1|bioproject)	-0.232	-0.501	0.041	0.793	0.606	1.042	ns
Oceania	Shannon	HIC	+log(readcount)	-0.169	-0.229	-0.111	0.844	0.796	0.895	*
Oceania	Shannon	HIC	+(1|bioproject)+ log(readcount)	-0.299	-0.561	-0.043	0.741	0.571	0.958	*
Oceania	ARG load	HIC	Reference	-0.449	-0.537	-0.361	0.638	0.585	0.697	*
Oceania	ARG load	HIC	+(1|bioproject)	-0.469	-0.975	0.026	0.626	0.377	1.026	ns
Oceania	ARG load	HIC	+log(readcount)	-0.462	-0.55	-0.375	0.63	0.577	0.687	*

Oceania	ARG load	HIC	+(1|bioproject)+ log(readcount)	-0.485	-0.981	0.009	0.616	0.375	1.009	ns
South America	Shannon	LMIC	Reference	-0.529	-0.654	-0.403	0.589	0.52	0.668	*
South America	Shannon	LMIC	+(1|bioproject)	-0.466	-1.269	0.31	0.628	0.281	1.364	ns
South America	Shannon	LMIC	+log(readcount)	-0.429	-0.571	-0.286	0.651	0.565	0.751	*
South America	Shannon	LMIC	+(1|bioproject)+ log(readcount)	-0.305	-1.216	0.519	0.737	0.296	1.68	ns
South America	ARG load	LMIC	Reference	-0.366	-0.544	-0.185	0.694	0.581	0.831	*
South America	ARG load	LMIC	+(1|bioproject)	-1.288	-3.011	0.116	0.276	0.049	1.123	ns
South America	ARG load	LMIC	+log(readcount)	-0.531	-0.748	-0.318	0.588	0.473	0.727	*
South America	ARG load	LMIC	+(1|bioproject)+ log(readcount)	-1.398	-3.105	0.093	0.247	0.045	1.097	ns
NA	Shannon	HIC	+log(readcount)	0.092	0.075	0.109	1.096	1.078	1.115	*
NA	Shannon	HIC	+(1|bioproject)+l og(readcount)	0.16	0.138	0.182	1.174	1.148	1.2	*
NA	Shannon	LMIC	+log(readcount)	0.125	0.037	0.212	1.134	1.038	1.236	*
NA	Shannon	LMIC	+(1|bioproject)+ log(readcount)	0.155	0.057	0.252	1.167	1.058	1.286	*
NA	ARG load	HIC	+log(readcount)	0.039	0.013	0.066	1.04	1.013	1.069	*
NA	ARG load	HIC	+(1|bioproject)+ log(readcount)	0.038	0.002	0.074	1.039	1.002	1.077	*
NA	ARG load	LMIC	+log(readcount)	-0.205	-0.336	-0.073	0.815	0.715	0.93	*
NA	ARG load	LMIC	+(1|bioproject)+ log(readcount)	-0.235	-0.381	-0.094	0.79	0.683	0.911	*

Supplementary Table 13. Drivers of antibiotic-specific ARG load in high-income countries (HIC) and low- and middle-income countries (LMIC). Probabilistic 95% credible intervals (CI) for the effect size of socio-economic variables on antibiotic-specific ARG load in a log-normal model. The five most prevalent antibiotic resistance classes were selected. The baseline categories for region are Europe and Asia in HIC and LMIC, respectively, and middle-aged adults for age. The effect sizes were mapped to percentage changes for easier interpretation, using the transform 100×(exp(*x*) - 1).

Predictor	Response	Q2.5	Q97.5	exp(Estimate)	exp(Q2.5)	exp(Q97.5)	Income Group
Intercept	Tetracycline	4.159	5.083	101.949	64.005	161.308	LMIC
Woman	Tetracycline	-0.13	0.282	1.077	0.878	1.326	LMIC
Europe	Tetracycline	-0.749	0.307	0.802	0.473	1.359	LMIC
South America	Tetracycline	-0.392	0.428	1.029	0.676	1.535	LMIC
Infant	Tetracycline	-3.162	-1.089	0.117	0.042	0.337	LMIC
Toddler	Tetracycline	-0.482	1.212	1.43	0.617	3.359	LMIC
Children	Tetracycline	-0.907	0.232	0.714	0.404	1.262	LMIC
Teenager	Tetracycline	-0.456	0.263	0.907	0.634	1.3	LMIC
Young Adult	Tetracycline	-0.421	0.193	0.894	0.656	1.213	LMIC
Older Adult	Tetracycline	-0.083	0.819	1.45	0.92	2.267	LMIC
Oldest Adult	Tetracycline	-0.47	1.334	1.561	0.625	3.795	LMIC
High Antibiotic Use	Tetracycline	-1.069	-0.261	0.51	0.343	0.77	LMIC
Intercept	Tetracycline	3.598	3.904	42.54	36.536	49.624	HIC
Woman	Tetracycline	0.185	0.437	1.366	1.203	1.548	HIC
Asia	Tetracycline	-0.18	1.997	2.461	0.835	7.366	HIC
North America	Tetracycline	-1.25	-0.897	0.342	0.287	0.408	HIC
Oceania	Tetracycline	0.089	0.674	1.47	1.093	1.963	HIC

Infant	Tetracycline	-3.07	-2.515	0.061	0.046	0.081	HIC
Toddler	Tetracycline	-0.704	0.002	0.701	0.495	1.002	HIC
Children	Tetracycline	-0.932	-0.516	0.483	0.394	0.597	HIC
Teenager	Tetracycline	-1.746	-1.274	0.22	0.174	0.28	HIC
Young Adult	Tetracycline	0.019	0.363	1.211	1.02	1.438	HIC
Older Adult	Tetracycline	-0.172	0.338	1.084	0.842	1.401	HIC
Oldest Adult	Tetracycline	-0.111	0.878	1.464	0.895	2.406	HIC
High Antibiotic Use	Tetracycline	0.148	0.5	1.387	1.16	1.648	HIC
Intercept	Beta-lactam	3.206	4.536	47.83	24.676	93.299	LMIC
Woman	Beta-lactam	-0.351	0.312	0.983	0.704	1.366	LMIC
Europe	Beta-lactam	0.366	1.974	3.26	1.442	7.2	LMIC
South America	Beta-lactam	-4.986	-3.728	0.013	0.007	0.024	LMIC
Infant	Beta-lactam	-1.807	0.994	0.686	0.164	2.703	LMIC
Toddler	Beta-lactam	-0.981	1.449	1.28	0.375	4.258	LMIC
Children	Beta-lactam	-0.507	1.186	1.404	0.603	3.273	LMIC
Teenager	Beta-lactam	-0.836	0.331	0.786	0.433	1.393	LMIC
Young Adult	Beta-lactam	-0.946	0.024	0.631	0.388	1.025	LMIC
Older Adult	Beta-lactam	-0.544	0.861	1.183	0.581	2.365	LMIC
Oldest Adult	Beta-lactam	-1.231	1.285	1.017	0.292	3.615	LMIC
High Antibiotic Use	Beta-lactam	-1.472	-0.208	0.43	0.229	0.812	LMIC
Intercept	Beta-lactam	2.529	2.912	15.22	12.544	18.392	HIC
Woman	Beta-lactam	-0.328	-0.01	0.843	0.72	0.99	HIC
Asia	Beta-lactam	-1.215	1.308	1.045	0.297	3.7	HIC

**North America**	**Beta-lactam**	**-0.903**	**-0.471**	**0.502**	**0.405**	**0.624**	**HIC**
**Oceania**	**Beta-lactam**	**-0.996**	**-0.268**	**0.526**	**0.369**	**0.765**	**HIC**
**Infant**	**Beta-lactam**	**-0.615**	**0.065**	**0.76**	**0.541**	**1.067**	**HIC**
**Toddler**	**Beta-lactam**	**0.455**	**1.316**	**2.413**	**1.576**	**3.73**	**HIC**
**Children**	**Beta-lactam**	**1.058**	**1.577**	**3.723**	**2.88**	**4.843**	**HIC**
**Teenager**	**Beta-lactam**	**1.026**	**1.61**	**3.735**	**2.789**	**5.001**	**HIC**
**Young Adult**	**Beta-lactam**	**-0.165**	**0.253**	**1.043**	**0.848**	**1.287**	**HIC**
**Older Adult**	**Beta-lactam**	**-0.558**	**0.095**	**0.792**	**0.572**	**1.1**	**HIC**
**Oldest Adult**	**Beta-lactam**	**-0.751**	**0.485**	**0.89**	**0.472**	**1.624**	**HIC**
**High Antibiotic Use**	**Beta-lactam**	**0.384**	**0.819**	**1.815**	**1.469**	**2.269**	**HIC**
**Intercept**	**Macrolide, Lincosamide, Streptogramin B**	**0.191**	**1.371**	**2.166**	**1.21**	**3.938**	**LMIC**
**Woman**	**Macrolide, Lincosamide, Streptogramin B**	**0.166**	**0.712**	**1.554**	**1.181**	**2.037**	**LMIC**
**Europe**	**Macrolide, Lincosamide, Streptogramin B**	**-3.143**	**-1.817**	**0.083**	**0.043**	**0.162**	**LMIC**
**South America**	**Macrolide, Lincosamide, Streptogramin B**	**-4.296**	**-3.201**	**0.024**	**0.014**	**0.041**	**LMIC**
**Infant**	**Macrolide, Lincosamide, Streptogramin B**	**-2.547**	**-0.073**	**0.271**	**0.078**	**0.93**	**LMIC**

Toddler	Macrolide, Lincosamide, Streptogramin B	0.276	2.422	3.824	1.317	11.27	LMIC
Children	Macrolide, Lincosamide, Streptogramin B	-0.518	0.949	1.234	0.596	2.584	LMIC
Teenager	Macrolide, Lincosamide, Streptogramin B	-0.37	0.604	1.132	0.691	1.829	LMIC
Young Adult	Macrolide, Lincosamide, Streptogramin B	0.088	0.929	1.667	1.092	2.531	LMIC
Older Adult	Macrolide, Lincosamide, Streptogramin B	-0.865	0.349	0.78	0.421	1.418	LMIC
Oldest Adult	Macrolide, Lincosamide, Streptogramin B	0.093	2.394	3.492	1.097	10.96	LMIC
High Antibiotic Use	Macrolide, Lincosamide, Streptogramin B	-0.64	0.442	0.908	0.527	1.555	LMIC
Intercept	Macrolide, Lincosamide, Streptogramin B	-2.711	-2.491	0.074	0.066	0.083	HIC
Woman	Macrolide, Lincosamide, Streptogramin B	-0.111	0.071	0.98	0.895	1.074	HIC
Asia	Macrolide, Lincosamide, Streptogramin B	1.497	3.198	10.571	4.467	24.474	HIC

North America	Macrolide, Lincosamide, Streptogramin B	-0.439	-0.179	0.736	0.645	0.836	HIC
Oceania	Macrolide, Lincosamide, Streptogramin B	-0.585	-0.155	0.692	0.557	0.856	HIC
Infant	Macrolide, Lincosamide, Streptogramin B	0.131	0.535	1.392	1.14	1.708	HIC
Toddler	Macrolide, Lincosamide, Streptogramin B	-0.556	-0.051	0.738	0.573	0.95	HIC
Children	Macrolide, Lincosamide, Streptogramin B	-0.319	-0.014	0.845	0.727	0.987	HIC
Teenager	Macrolide, Lincosamide, Streptogramin B	-0.491	-0.153	0.726	0.612	0.858	HIC
Young Adult	Macrolide, Lincosamide, Streptogramin B	-0.204	0.05	0.923	0.815	1.052	HIC
Older Adult	Macrolide, Lincosamide, Streptogramin B	0.118	0.504	1.358	1.125	1.655	HIC
Oldest Adult	Macrolide, Lincosamide, Streptogramin B	-0.053	0.706	1.385	0.948	2.025	HIC
High Antibiotic Use	Macrolide, Lincosamide, Streptogramin B	0.222	0.487	1.423	1.249	1.628	HIC
Intercept	Aminoglycoside	-1.5	-0.033	0.459	0.223	0.968	LMIC

Woman	Aminoglycoside	-0.513	0.241	0.868	0.599	1.272	LMIC
Europe	Aminoglycoside	0.934	2.605	5.805	2.544	13.528	LMIC
South America	Aminoglycoside	-0.123	1.245	1.764	0.885	3.473	LMIC
Infant	Aminoglycoside	0.218	3.009	5.011	1.243	20.268	LMIC
Toddler	Aminoglycoside	-1.211	1.349	1.043	0.298	3.853	LMIC
Children	Aminoglycoside	-0.671	1.127	1.274	0.511	3.086	LMIC
Teenager	Aminoglycoside	-0.698	0.551	0.924	0.498	1.735	LMIC
Young Adult	Aminoglycoside	-0.923	0.172	0.688	0.397	1.188	LMIC
Older Adult	Aminoglycoside	-0.672	0.901	1.123	0.511	2.463	LMIC
Oldest Adult	Aminoglycoside	-0.584	2.078	2.096	0.557	7.986	LMIC
High Antibiotic Use	Aminoglycoside	-0.158	1.287	1.769	0.853	3.623	LMIC
Intercept	Aminoglycoside	-1.781	-1.429	0.201	0.168	0.24	HIC
Woman	Aminoglycoside	0.443	0.728	1.798	1.557	2.072	HIC
Asia	Aminoglycoside	-0.219	2.25	2.734	0.804	9.485	HIC
North America	Aminoglycoside	-1.562	-1.16	0.257	0.21	0.313	HIC
Oceania	Aminoglycoside	-0.894	-0.207	0.574	0.409	0.813	HIC
Infant	Aminoglycoside	-0.253	0.386	1.067	0.776	1.471	HIC
Toddler	Aminoglycoside	-0.797	0.015	0.677	0.451	1.015	HIC
Children	Aminoglycoside	-1.306	-0.828	0.343	0.271	0.437	HIC
Teenager	Aminoglycoside	-1.491	-0.944	0.295	0.225	0.389	HIC
Young Adult	Aminoglycoside	-0.111	0.291	1.09	0.895	1.337	HIC
Older Adult	Aminoglycoside	0.788	1.391	2.986	2.2	4.02	HIC
Oldest Adult	Aminoglycoside	1.313	2.47	6.609	3.718	11.819	HIC

High Antibiotic Use	Aminoglycoside	1.223	1.628	4.168	3.397	5.094	HIC
Intercept	Amphenicol	-3.329	-1.92	0.073	0.036	0.147	LMIC
Woman	Amphenicol	0.171	0.852	1.667	1.187	2.346	LMIC
Europe	Amphenicol	1.583	3.131	10.603	4.868	22.888	LMIC
South America	Amphenicol	0.514	1.791	3.195	1.672	5.994	LMIC
Infant	Amphenicol	-0.77	2.027	1.833	0.463	7.595	LMIC
Toddler	Amphenicol	-1.313	1.133	0.913	0.269	3.105	LMIC
Children	Amphenicol	0.153	1.866	2.68	1.165	6.459	LMIC
Teenager	Amphenicol	-0.315	0.868	1.31	0.73	2.383	LMIC
Young Adult	Amphenicol	0.076	1.094	1.788	1.079	2.987	LMIC
Older Adult	Amphenicol	0.175	1.61	2.447	1.192	5.003	LMIC
Oldest Adult	Amphenicol	-0.647	1.825	1.761	0.524	6.203	LMIC
High Antibiotic Use	Amphenicol	-0.065	1.25	1.828	0.937	3.49	LMIC
Intercept	Amphenicol	-0.412	-0.088	0.778	0.662	0.915	HIC
Woman	Amphenicol	-0.164	0.094	0.965	0.848	1.098	HIC
Asia	Amphenicol	-0.544	1.664	1.764	0.581	5.282	HIC
North America	Amphenicol	-2.884	-2.511	0.067	0.056	0.081	HIC
Oceania	Amphenicol	-2.015	-1.404	0.182	0.133	0.246	HIC
Infant	Amphenicol	-1.736	-1.155	0.237	0.176	0.315	HIC
Toddler	Amphenicol	-0.548	0.161	0.826	0.578	1.174	HIC
Children	Amphenicol	-0.452	-0.02	0.788	0.636	0.98	HIC
Teenager	Amphenicol	-0.688	-0.209	0.639	0.503	0.811	HIC

**Young Adult**	**Amphenicol**	**-0.041**	**0.317**	**1.148**	**0.96**	**1.373**	**HIC**
**Older Adult**	**Amphenicol**	**0.623**	**1.16**	**2.435**	**1.865**	**3.189**	**HIC**
**Oldest Adult**	**Amphenicol**	**1.004**	**2.068**	**4.627**	**2.729**	**7.911**	**HIC**
**High Antibiotic Use**	**Amphenicol**	**0.982**	**1.363**	**3.237**	**2.669**	**3.91**	**HIC**
